# NKp46 enhances type 1 innate lymphoid cell proliferation and function and anti-acute myeloid leukemia activity

**DOI:** 10.1038/s41467-025-55923-w

**Published:** 2025-01-24

**Authors:** Rui Ma, Zhenlong Li, Hejun Tang, Xiaojin Wu, Lei Tian, Zahir Shah, Ningyuan Liu, Tasha Barr, Jianying Zhang, Sean Wang, Srividya Swaminathan, Guido Marcucci, Yong Peng, Michael A. Caligiuri, Jianhua Yu

**Affiliations:** 1 Center for Molecular Oncology, State Key Laboratory of Biotherapy and Cancer Center, West China Hospital, Sichuan University, Chengdu, 610041 China; 2 Department of Hematology & Hematopoietic Cell Transplantation, City of Hope National Medical Center, Los Angeles, CA 91010 USA; 3 The First Affiliated Hospital of Soochow University, Suzhou, 215005 China; 4 Division of Hematology & Oncology, Department of Medicine, School of Medicine, University of California, Irvine, CA 92697 USA; 5 The Clemons Family Center for Transformative Cancer Research, University of California, Irvine, CA 92697 USA; 6 Department of Computational and Quantitative Medicine, City of Hope National Medical Center, Los Angeles, CA 91010 USA; 7 Division of Transfusion Medicine, City of Hope National Medical Center, Los Angeles, CA 91010 USA; 8 Department of Systems Biology, City of Hope Beckman Research Institute, Los Angeles, CA 91010 USA; 9 Gehr Family Center for Leukemia Research, Hematologic Malignancies Research Institute, Department of Hematological Malignancies Translational Science, City of Hope National Medical Center, Los Angeles, CA 91010 USA

**Keywords:** Innate lymphoid cells, Immunosurveillance, Cell death and immune response

## Abstract

NKp46 is a critical regulator of natural killer (NK) cell immunity, but its function in non-NK innate immune cells remains unclear. Here, we show that NKp46 is indispensable for expressing IL-2 receptor-α (IL-2Rα) by non-NK liver-resident type-1 innate lymphoid cells (ILC1s). Deletion of NKp46 reduces IL-2Rα on ILC1s by downregulating NF-κB signaling, thus impairing ILC1 proliferation and cytotoxicity in vitro and in vivo. The binding of anti-NKp46 antibody to NKp46 triggers the activation of NF-κB, the expression of IL-2Rα, interferon-γ (IFN-γ), tumor necrosis factor (TNF), proliferation, and cytotoxicity. Functionally, NKp46 expressed on mouse ILC1s interacts with tumor cells through cell–cell contact, increasing ILC1 production of IFN-γ and TNF, and enhancing cytotoxicity. In a mouse model of acute myeloid leukemia, deletion of NKp46 impairs the ability of ILC1s to control tumor growth and reduces survival. This can be reversed by injecting NKp46^+^ ILC1s into NKp46 knock-out mice. Human NKp46^+^ ILC1s exhibit stronger cytokine production and cytotoxicity than their NKp46^−^ counterparts, suggesting that NKp46 plays a similar role in humans. These findings identify an NKp46–NF-κB–IL-2Rα axis and suggest that activating NKp46 with an anti-NKp46 antibody may provide a potential strategy for anti-tumor innate immunity.

## Abstract (web summary)

It has been previously reported that the natural cytotoxic receptor NKp46 plays an important role in regulating natural killer (NK) cell biology. Here the authors show that NKp46 is required for the proliferation and cytotoxicity of type 1 innate lymphoid cells (ILCs) by activating the NF-κB signaling pathway and regulating IL-2Rα expression.

## Introduction

Innate lymphoid cells (ILCs) are tissue-resident innate immune cells that respond rapidly to infection and secrete inflammatory mediators similar to those from T lymphocytes^1^. Unlike T lymphocytes, ILCs do not express antigen-specific receptors that require recombination-activating gene (RAG)-dependent rearrangement. Nevertheless, both types of immune cells express subunits of cytokine receptors, including the interleukin (IL)-2 receptor-α and IL-7 receptor-α (IL-7Rα or CD127)^2–4^. Therefore, ILCs are considered the innate immune system’s immunoregulatory counterparts to CD4 lymphocytes^1^. ILCs can be classified into three subgroups, namely groups 1–3, based on their cytokine profiles and transcription factor requirements during development^2^. Group 1 includes ILC1s and natural killer (NK) cells. In mice, both cell types produce interferon-gamma (IFN-γ) and require T-bet for their function^5^. While human and mouse NK cells also express the transcription factor eomesodermin (Eomes) and have high levels of cytotoxicity, ILC1s do not express Eomes in mice and have low levels of cytotoxicity^6^. Human peripheral blood ILC1s express CD127, which distinguishes them from cytotoxic CD16^+^CD56^+^ NK cells that lack this marker^4^. Group 2, known as ILC2s, express GATA3 and secrete IL-4, IL-5, IL-9, IL-13, and arginase-1^7^. Group 3 consists of ILC3s and expresses RAR-related orphan receptor gamma t (RORγt) and produces IL-22 and IL-17^8,9^.

ILC1s develop from common helper-like ILC progenitors (CHILPs) through innate lymphoid cell precursor (ILCP) cells, which can be detected before birth^10–13^. ILC1s function to rapidly protect the host from bacterial and viral pathogens at initial sites of infection primarily through producing the cytokines IFN-γ and TNF^14–19^. The role of transforming growth factor β (TGF-β) is pivotal in the plasticity of ILC1s^20,21^. Notably, SMAD family member 4 (SMAD4) blocks the conversion of NK cells into ILC1-like cells by curtailing non-canonical TGF-β signaling^22,23^. Multiple studies have also shown that ILC1s play critical roles in tumor surveillance and tumorigenesis. For example, they are essential in the defense against liver metastasis^24–27^. However, the mechanisms through which ILC1s target tumor cells are mostly undefined.

Natural cytotoxic receptor NKp46, encoded by the *Ncr1* gene, is a member of the natural cytotoxicity receptor (NCR) family. In most situations, it plays an important role in regulating NK cell recognition and clearing virus-infected cells and tumor cells^28^. In mice, non-NK immune cells also express NKp46, including ILC1s (defined as Lin^−^NK1.1^+^NKp46^+^CD49a^+^CD49b^−^) and some ILC3s (identified as Lin^−^CD127^+^NKp46^+^RORγt^+^)^24^. However, the role of NKp46 in these non-NK immune cells remains largely unknown. We and others have previously reported that NKp46 is required for surface expression of TNF-related apoptosis-inducing ligand (TRAIL) on ILC1s^29–31^. Additionally, NKp46 deficiency notably reduces both the percentages and numbers of ILC1s in mice^30^. By contrast, wild-type (WT) and NKp46-deficient mice have similar proportions of NK cells, ILC2s, and ILC3s. This suggests that NKp46 has a distinct and specific role in the regulation of ILC1s^30^. However, the precise mechanism by which NKp46 exerts its regulatory effects on ILC1s remains to be explored.

In this work, we demonstrate that NKp46 is required for the proliferation of ILC1s by regulating IL-2Rα expression. The binding of anti-NKp46 antibody to NKp46 triggers the expression of IL-2Rα by activating nuclear factor (NF)-κB signaling and induces the production of IFN-γ and TNF. This engagement also enhances NKp46-mediated cytotoxicity in ILC1s. NKp46 deficiency in a mouse model of AML reduces the ability to control leukemic cells and compromises survival. This defect can be reversed by injecting enough NKp46^+^ ILC1s into NKp46-deficient mice. Finally, human NKp46^+^ ILC1s exhibit enhanced cytokine production and cytotoxic activity compared with their NKp46^–^ counterparts, indicating that NKp46 likely serves similar functions in both humans and mice.

## Results

### NKp46 deficiency downregulates IL-18R1, IL-7Rα, and IL-2Rα expression on ILC1s

**Fig. 1:**
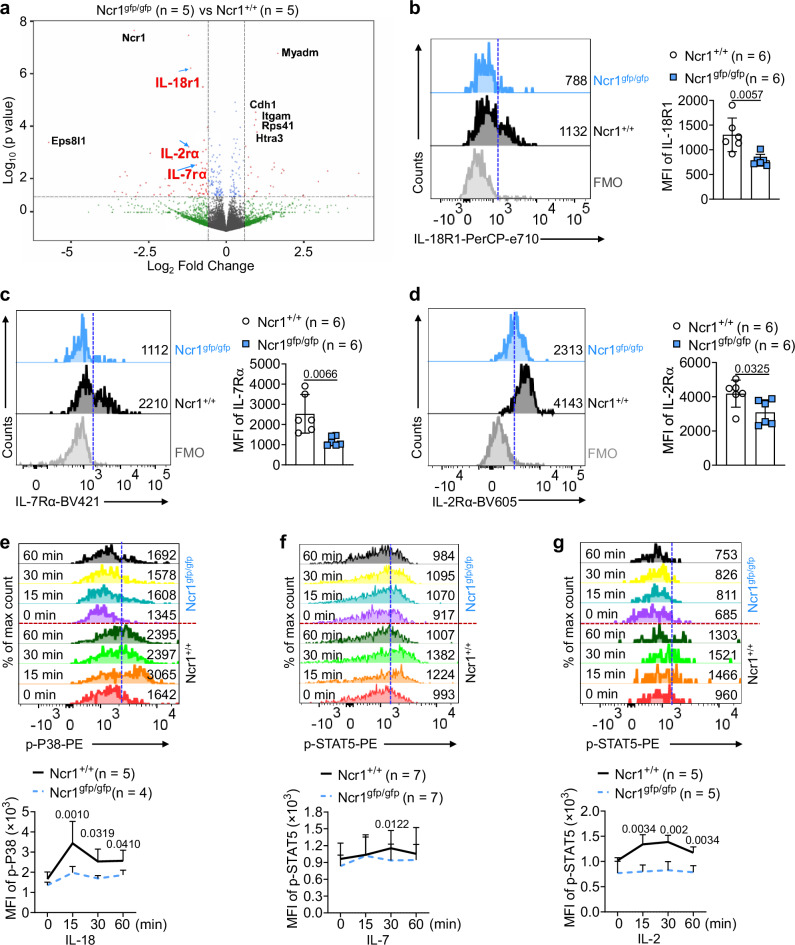
NKp46 supports IL-18R1, IL-7Rα, and IL-2Rα expression on ILC1s. **a** *Ncr1*^*+/+*^ and *Ncr1*^*gfp/gfp*^ ILC1s were sorted for RNA-seq from the liver of *Ncr1*^*+/+*^ mice and *Ncr1*^*gfp/gfp*^ mice, respectively, using FACS. Volcano plots show statistically significant differentially expressed genes in RNA pools (*Ncr1*^*gfp/gfp*^ ILC1s vs. *Ncr1*^*+/+*^ ILC1s) (*n* = 5). **b**–**d** Expression of IL-18R1 (**b**), IL-7Rα (**c**), and IL-2Rα (**d**) on the liver *Ncr1*^*+/+*^ ILC1s and *Ncr1*^*gfp/gfp*^ ILC1s was measured by flow cytometry (*n* = 6). **e** *Ncr1*^*+/+*^ ILC1s and *Ncr1*^*gfp/gfp*^ ILC1s were treated with IL-18 (10 ng/ml) at the indicated time points. Phosphorylation of P38 was measured by flow cytometry (*Ncr1*^*+/+*^: *n* = 5; *Ncr1*^*gfp/gfp*^: *n* = 4). **f**, **g** *Ncr1*^*+/+*^ ILC1s and *Ncr1*^*gfp/gfp*^ ILC1s were treated with IL-7 (100 ng/ml; **f**) or IL-2 (1000 IU/ml; **g**) at the indicated time points. Phosphorylation of STAT5 was measured by flow cytometry (**f**: *n* = 7; **g**: *n* = 5). Data were presented as means ± s.d.; *P* values were calculated by either a two-tailed Student’s *t*-test (**b**–**d** and **f** at 30 min) or linear mixed models with adjustments (**e**, **g**). Source data are provided as a Source Data file.

To determine how NKp46 regulates ILC1 development and function, we isolated freshly sorted ILC1s from the livers of *Ncr1*^*+/+*^ and *Ncr1*^*gfp/gfp*^ mice and performed RNA sequencing (RNA-seq). RNA-seq analysis identified 35 upregulated genes and 66 downregulated genes in *Ncr1*^*gfp/gfp*^ ILC1s as compared to *Ncr1*^*+/+*^ ILC1s (false discovery rate (FDR) adjusted *P* < 0.05 and Log_2_FC > 1) (Supplementary Fig. 1a, b and Supplementary Fig. 2a, b). Among the genes that were differentially expressed, we noted decreased expression of the cytokine receptors *Il-18r1*, *Il-7rα*, and *Il-2rα* in *Ncr1*^*gfp/gfp*^ ILC1s compared to *Ncr1*^*+/+*^ ILC1s (Fig. 1a). The downregulation of cytokine receptors *Il-18r1*, *Il-7rα*, and *Il-2rα* was confirmed at the protein level by flow cytometry, which demonstrated that *Ncr1*^*gfp/gfp*^ ILC1s had significantly lower expression of IL-18R1, IL-7Rα, and IL-2Rα compared to *Ncr1*^*+/+*^ ILC1s (Fig. 1b–d). In contrast to these results, expression of IL-15 receptors (CD122 and CD132) and IL-12 receptors (IL-12Rβ1 and IL-12Rβ2) were comparable between *Ncr1*^*+/+*^ ILC1s and *Ncr1*^*gfp/gfp*^ ILC1s (Supplementary Fig. 3a, b). Notably, the expression of IL-15Rα (CD215) was higher on *Ncr1*^*gfp/gfp*^ ILC1s compared to *Ncr1*^*+/+*^ ILC1s. To further validate the functional implications of the reduced expression of IL-18R1, IL-7Rα, and IL-2Rα, we administered their respective cytokines—IL-18, IL-7, and IL-2—to both *Ncr1*^*+/+*^ ILC1s and *Ncr1*^*gfp/gfp*^ ILC1s. Subsequently, we assessed the phosphorylation of P38, which is one of the downstream targets of IL-18R1, and STAT5, which is a downstream target of IL-7Rα and IL-2Rα^32–34^. *Ncr1*^*gfp/gfp*^ ILC1s exhibited significantly lower levels of P38 phosphorylation than *Ncr1*^*+/+*^ ILC1s upon stimulation with IL-18 (Fig. 1e). Similarly, phosphorylation of STAT5 in *Ncr1*^*gfp/gfp*^ ILC1s was also significantly less than in *Ncr1*^*+/+*^ ILC1s following treatment with IL-7 or IL-2 (Fig. 1f, g), suggesting that NKp46 deficiency decreases the response of ILC1s to these cytokines. Interestingly, this disparity between the two subsets of ILC1s was not evident in the production of IFN-γ or TNF, either under resting conditions or following IL-2 or IL-18 stimulation (Supplementary Fig. 3c). This could be due to low levels of IFN-γ or TNF production induced by single cytokine stimulation with IL-2 or IL-18, as previously demonstrated in NK cells, where NKp46 knock-out did not result in reduced IFN-γ production either under various stimulations^35,36^. These findings demonstrate that the production of IFN-γ or TNF by ILC1s is not effectively triggered by a single cytokine corresponding to IL-2Rα or IL-18R1. We also evaluated the expression of granzymes (Gzms) A, B, and C in both groups of mice. The expression of GzmB and GzmC was significantly reduced in *Ncr1*^*gfp/gfp*^ ILC1s compared to *Ncr1*^*+/+*^ ILC1s, suggesting a potential impairment in their cytotoxic functions (Supplementary Fig. 3d). By contrast, GzmA expression remained comparable between the two groups (Supplementary Fig. 3d), indicating that not all granzyme profiles are affected by absence of NKp46. Collectively, these results suggest that NKp46 is involved in regulating select cytokine receptor expression on ILC1s, as well as the expression of GzmB and GzmC.

### Anti-NKp46 antibody triggers activation of the NF-κB signaling pathway in ILC1s

**Fig. 2:**
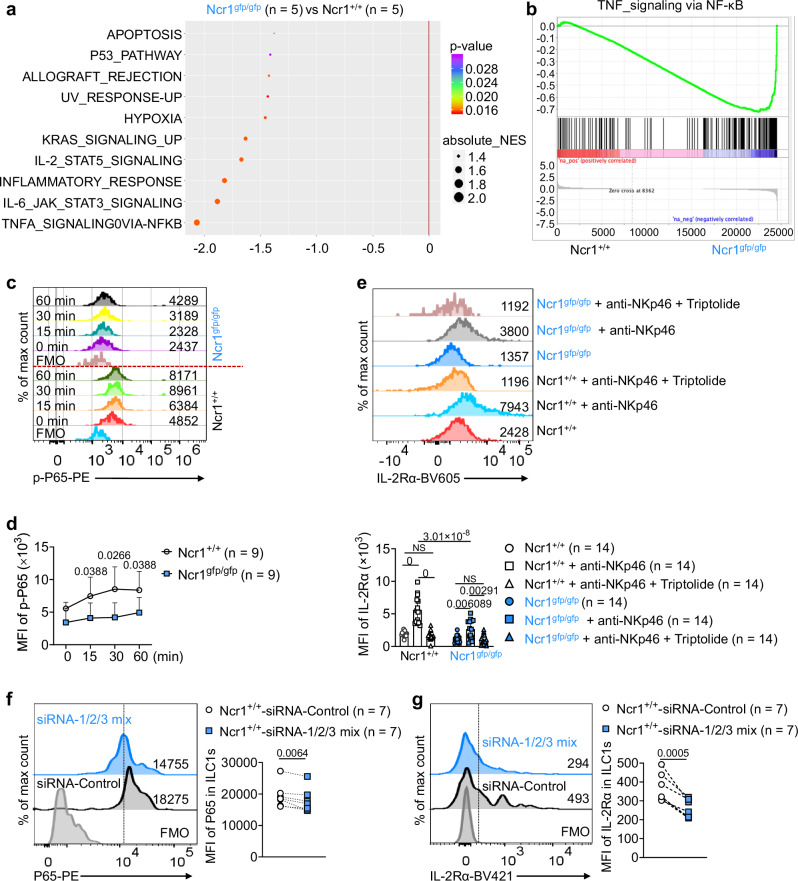
NKp46-triggered signaling activates the NF-κB signaling pathway and IL-2Rα expression. **a** Hallmark pathway analysis in *Ncr1*^*+/+*^ ILC1s and *Ncr1*^*gfp/gfp*^ ILC1s RNA pools (*Ncr1*^*gfp/gfp*^ ILC1s vs. *Ncr1*^*+/+*^ ILC1s). The panel shows signaling pathways downregulated in *Ncr1*^*gfp/gfp*^ ILC1s. Genes with a false discovery rate (FDR)-adjusted *P* value < 0.05 and a fold change (FC) > 1.5 or < 0.7 were significantly upregulated or downregulated, respectively (*n* = 5). **b** Gene set enrichment analysis (GSEA) plots show enrichment of the TNF gene in the NF-κB signaling pathway in ILC1s. The x-axis shows the rank orders (*Ncr1*^*gfp/gfp*^ ILC1s vs. *Ncr1*^*+/+*^ ILC1s) of all the targeted genes (*n* = 5). **c**, **d** *Ncr1*^*+/+*^ ILC1s and *Ncr1*^*gfp/gfp*^ ILC1s were treated with or without anti-NKp46 antibody (5 μg/ml) for the indicated time. Phosphorylation of P65 (p-P65) was measured by flow cytometry. Representative histogram (**c**) and quantification of p-P65 mean fluorescence intensity (MFI) (**d**) in *Ncr1*^*+/+*^ ILC1s and *Ncr1*^*gfp/gfp*^ ILC1s at the indicated time points (*n* = 9). FMO: fluorescence minus one. Data from two experiments were pooled. **e** *Ncr1*^*+/+*^ ILC1s and *Ncr1*^*gfp/gfp*^ ILC1s were treated with anti-NKp46 antibody (5 μg/ml) in the presence or absence of NF-κB inhibitor (Triptolide, 10 nM) for 3 days. Representative histogram of (top) and quantification (bottom) of IL-2Rα MFI on *Ncr1*^*+/+*^ ILC1s and *Ncr1*^*gfp/gfp*^ ILC1s (*n* = 14). **f** ILC1s were transfected with control silence RNA (siRNA-control) or siRNA (siRNA-1/2/3 mix) for interfering P65 for 3 days using lipofectamine 3000. Histogram (left) and statistics graphic (right) showing the expression of P65 in ILC1s (*n* = 7). **g** ILC1s were transfected with control siRNA or siRNA (siRNA-1/2/3 mix) for interfering P65 for 3 days using lipofectamine 3000. Histogram (left) and statistics graphic (right) showing the expression of IL-2Rα in ILC1s (*n* = 7). Data were presented as means ± s.d.; *P* values were calculated by linear mixed models with adjustments (**d**), one-way ANOVA models with adjustments (**e**), or a paired two-tailed Student’s *t*-test (**f**, **g**). NS not significant. Source data are provided as a Source Data file.

Using gene set enrichment analysis (GSEA) of RNA-seq data from freshly sorted ILC1s (Lin^*−*^NK1.1^+^NKp46^+^CD49b^*−*^CD49a^+^) from the livers of *Ncr1*^*+/+*^ and *Ncr1*^*gfp/gfp*^ mice, we identified the top ten pathways associated with downregulated genes. Notably, NKp46 deletion significantly affected the NF-κB and Janus kinase-signal transducer and activator of transcription (JAK/STAT) 3/5 signaling pathways (Fig. 2a, b and Supplementary Fig. 4a). To validate these findings, we treated ILC1s with an anti-NKp46 antibody and found that the phosphorylation of NF-κB P65 subunit was increased in *Ncr1*^*+/+*^ ILC1s compared to *Ncr1*^*gfp/gfp*^ ILC1s (Fig. 2c, d), while the phosphorylation of STAT3/5 was not (Supplementary Fig. 4b, c). Moreover, when we treated *Ncr1*^*+/+*^ NK cells and *Ncr1*^*gfp/gfp*^ NK cells with anti-NKp46 antibody, the differential phosphorylation of NF-κB p65 was not observed (Supplementary Fig. 4d). Taken together, these data suggest that the engagement of NKp46 selectively activates the NF-κB signaling pathway in liver ILC1s but not in liver NK cells. Contrasting with the effects of cytokine activation (Fig. 1f, g), NKp46 engagement does not activate the JAK/STAT3/5 signaling pathway in liver ILC1s.

### Stimulation of NF-κB activity by anti-NKp46 antibody augments IL-2Rα expression on ILC1s

We next hypothesized that NKp46 regulates IL-18R1, IL-7Rα, and IL-2Rα expression through the NF-κB signaling pathway. As expected, our data showed that expression of IL-2Rα was upregulated on *Ncr1*^*+/+*^ ILC1s following stimulation with anti-NKp46 antibody (Fig. 2e). Interestingly, while upregulation of IL-2Rα was also observed in *Ncr1*^*gfp/gfp*^ ILC1s, the levels of IL-2Rα following anti-NKp46 antibody treatment remained significantly lower compared to those in *Ncr1*^*+/+*^ ILC1s (Fig. 2e). Blockade of the NF-κB signaling pathway with an optimized concentration of Triptolide (an NF-κB inhibitor; Supplementary Fig. 4e) suppressed this upregulation induced by engagement with NKp46 (Fig. 2e). However, the STAT3 inhibitor C188-9 and the STAT5 inhibitor STAT5-IN-1 did not block the induction of IL-2Rα in *Ncr1*^*+/+*^ ILC1s by anti-NKp46 antibody (Supplementary Fig. 4f). We then used P65 silencing RNA to knock down P65 in the ILC1s and observed a decrease in IL-2Rα expression compared to the control group, where P65 was not knocked down (Fig. 2f, g). In addition, in vitro stimulation of NKp46 with anti-NKp46 antibody did not directly affect the expression of IL-18R1 and IL-7Rα on ILC1s, irrespective of the presence or absence of Triptolide (Supplementary Fig. 4g, h). Collectively, these data indicate that activation via NKp46 induces the expression of IL-2Rα on ILC1s by activating NF-κB rather than by activating the JAK/STAT3/5 signaling pathway.

### NKp46 deletion decreases the proliferation of ILC1s

**Fig. 3:**
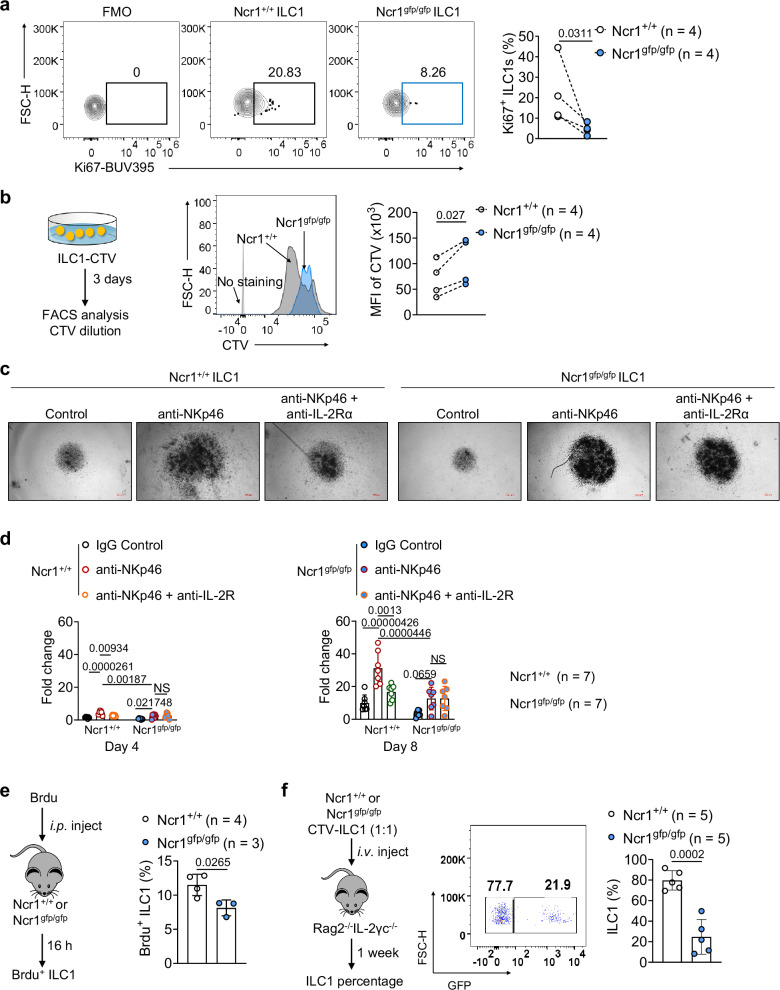
NKp46 deletion decreases ILC1 proliferation in vitro and in vivo. **a** Percentages of Ki67^+^ ILC1s in the liver of *Ncr1*^*+/+*^ and *Ncr1*^*gfp/gfp*^ mice were measured by flow cytometry (*n* = 4). **b** *Ncr1*^*+/+*^ ILC1s and *Ncr1*^*gfp/gfp*^ ILC1s were sorted from *Ncr1*^*+/+*^ and *Ncr1*^*gfp/gfp*^ mice, respectively, labeled with cell trace violet (CTV), and then were cultured with IL-2 (1000 IU/ml) and IL-7 (100 ng/ml) for 3 days. Quantification of CTV dilution in *Ncr1*^*+/+*^ ILC1s and *Ncr1*^*gfp/gfp*^ ILC1s (*n* = 4). **c**, **d** *Ncr1*^*+/+*^ ILC1s and *Ncr1*^*gfp/gfp*^ ILC1s sorted from the liver of *Ncr1*^*+/+*^ and *Ncr1*^*gfp/gfp*^ mice, respectively, were cultured with or without isotype antibody (5 μg/ml; Control) or anti-NKp46 antibody (5 μg/ml) in the presence or absence of anti-IL-2Rα (20 μg/ml) for indicated days, in a medium containing IL-2 (1000 IU/ml) and IL-7 (100 ng/ml). Representative images (**c**: scale bar, 500 µm) on day 8 and statistics (**d**) of the fold change in ILC1 numbers on day 4 and day 8 (*n* = 7). **e** *Ncr1*^*+/+*^ or *Ncr1*^*gfp/gfp*^ mice were i.p. injected with 200 µl of BrdU solution. Sixteen hours later, BrdU^+^ ILC1s in the liver of *Ncr1*^*+/+*^ and *Ncr1*^*gfp/gfp*^ mice were measured by flow cytometry (*Ncr1*^*+/+*^ mice: *n* = 4; *Ncr1*^*gfp/gfp*^ mice: *n* = 3). **f** *Ncr1*^*+/+*^ (GFP-negative) or *Ncr1*^*gfp/gfp*^ (GFP-positive) ILC1s were sorted from the liver of mice, mixed at a ratio of 1:1, and then i.v. injected into *Rag2*^***−****/****−***^*γc*^***−/−***^mice. One week later, percentages of *Ncr1*^*+/+*^ ILC1s and *Ncr1*^*gfp/gfp*^ ILC1s in the liver of mice were measured by flow cytometry (*n* = 5). Data were presented as means ± s.d.; *P* values were calculated by a paired two-tailed Student’s *t-*test (**a**, **b**), an unpaired two-tailed Student’s *t*-test (**e**, **f**), or one-way ANOVA models with adjustments (**d**). NS not significant. Source data are provided as a Source Data file.

Memory CD8^+^ T cells (T_M_ cells), CD4^+^ T_M_ cells, and regulatory T cells express higher levels of *Il2rα* and *Il7rα* than naïve T cells^15,37^. T_M_ cells require IL-7 for their survival and persistence during homeostasis^38,39^, while human CD56^bright^ NK cells have a survival and proliferative response to IL-2^40,41^. Thus, the downregulation of IL-2Rα and IL-7Rα on *Ncr1*^*gfp/gfp*^ ILC1s led us to investigate whether NKp46 also regulates ILC1 proliferation, survival, or persistence. We observed comparable levels of apoptosis in ILC1s freshly isolated from *Ncr1*^*+/+*^ and *Ncr1*^*gfp/gfp*^ mice (Supplementary Fig. 5a–c), as determined by caspase 3 and annexin V staining—two hallmarks of apoptotic cell death. Thus, the lack of NKp46 does not affect ILC1 apoptosis. However, expression of the proliferation marker Ki67 was significantly downregulated in freshly isolated *Ncr1*^*gfp/gfp*^ ILC1s compared to *Ncr1*^*+/+*^ ILC1s (Fig. 3a). We also used a cell proliferation tracing dye (cell trace violet, CTV) to label ILC1s sorted from the liver of *Ncr1*^*+/+*^ and *Ncr1*^*gfp/gfp*^ mice and then cultured them with IL-2 and IL-7 for 3 days. CTV was less diluted in the *Ncr1*^*gfp/gfp*^ ILC1 cultures than in the *Ncr1*^*+/+*^ ILC1 cultures, confirming less proliferation (Fig. 3b). In addition, the fold increase in the number of *Ncr1*^*gfp/gfp*^ ILC1s stimulated with IL-2 and IL-7 was significantly lower than that of *Ncr1*^*+/+*^ ILC1s, regardless of the presence or absence of anti-NKp46 antibody (Supplementary Fig. 5d). This correlated with increased IL-2Rα expression (Supplementary Fig. 5e). However, in both subsets, IL-7Rα was almost undetectable after co-culturing cells with IL-2 and IL-7 (Supplementary Fig. 5f). These observations are consistent with a previous report that IL-7 or IL-2 dramatically suppresses the expression of IL-7Rα on T cells and ILCs^42,43^. When IL-2Rα was blocked with an anti-IL-2Rα antibody, the growth of *Ncr1*^*+/+*^ ILC1s was inhibited under culture conditions with IL-2, IL-7, and the anti-NKp46 antibody (Fig. 3c, d). This effect was not observed in the *Ncr1*^*gfp/gfp*^ ILC1s. Collectively, these data suggest that NKp46 stimulation is necessary for ILC1 proliferation in the presence of IL-2 because it induces those cells to express IL-2Rα. In the experiments supplemented with an anti-NKp46 antibody, we observed an interesting phenomenon where the anti-NKp46 antibody also elicited a response from *Ncr1*^*gfp/gfp*^ ILC1s, resulting in an increase in ILC1 cell number in the culture compared to those without the anti-NKp46 antibody (Supplementary Fig. 5d). We then examined the NKp46 expression on ILC1s isolated from the liver of *Ncr1*^*gfp/gfp*^ mice. Negligible expression of NKp46 was observed, with only an average of 3.25% of cells being NKp46 positive among *Ncr1*^*gfp/gfp*^ ILC1s (Supplementary Fig. 5g). As shown in Fig. 3d, the proliferation of *Ncr1*^*gfp/gfp*^ ILC1s induced by the anti-NKp46 antibody remained unaltered compared to cultures with a blocking antibody to IL-2Rα, suggesting that this response is not related to IL-2Rα. It is noteworthy that there are approximately 3.25% of NKp46^+^ ILC1 cells among *Ncr1*^*gfp/gfp*^ ILC1s, which assumably is contributing to the relative response of *Ncr1*^*gfp/gfp*^ ILC1s to the anti-NKp46 antibody (Supplementary Fig. 5g). Importantly, when comparing *Ncr1*^*gfp/gfp*^ ILC1s to *Ncr1*^*+/+*^ ILC1s stimulated with the anti-NKp46 antibody, the proliferation of *Ncr1*^*gfp/gfp*^ ILC1s was significantly reduced under the same conditions. Thus, it seems evident that the proliferation of ILC1s is associated with NKp46.

To determine whether NKp46 also influences ILC1 proliferation in vivo, we intraperitoneally (i.p.) injected *Ncr1*^*+/+*^ and *Ncr1*^*gfp/gfp*^ mice with bromodeoxyuridine (BrdU) and analyzed the frequency of BrdU^+^ ILC1s in the liver 16 h post-injection. We observed significantly fewer BrdU^+^ ILC1s in the liver of *Ncr1*^*gfp/gfp*^ mice than in that of *Ncr1*^*+/+*^ mice (Fig. 3e). We next intravenously (i.v.) co-transferred equal numbers of FACS-sorted *Ncr1*^*+/+*^ and *Ncr1*^*gfp/gfp*^ ILC1s labeled with CTV (to distinguish donor cells from host cells) into immunodeficient recipient *Rag2*^−/−^*Il2γc*^−/−^ mice, which lack T, B, ILCs, and NK cells. Of note, CD200R was previously found to be expressed on liver ILC1s but not liver NK cells^14^. We, therefore, assessed the expression of the CD200R and NK1.1 markers in CTV^+^ cells. We found that all CTV^+^ cells were confirmed to be genuine ILC1s expressing CD200R and NK1.1, and they had not converted into NK cells. We next used GFP to distinguish *Ncr1*^*+/+*^ (GFP^−^) and *Ncr1*^*gfp/gfp*^ (GFP^+^) ILC1s in the experiment (Supplementary Fig. 5h). After 1 week, we examined the ratio change between GFP^+^ and GFP^−^ cells and found the population of *Ncr1*^*gfp/gfp*^ ILC1s was less than those of *Ncr1*^*+/+*^ ILC1s in the recipient *Rag2*^−/−^*Il2γc*^−/−^ mice, confirming that full ILC1 proliferation requires NKp46 in vivo (Fig. 3f).

### NKp46 deletion impairs ILC1 cytokine production and cytotoxicity against AML

**Fig. 4:**
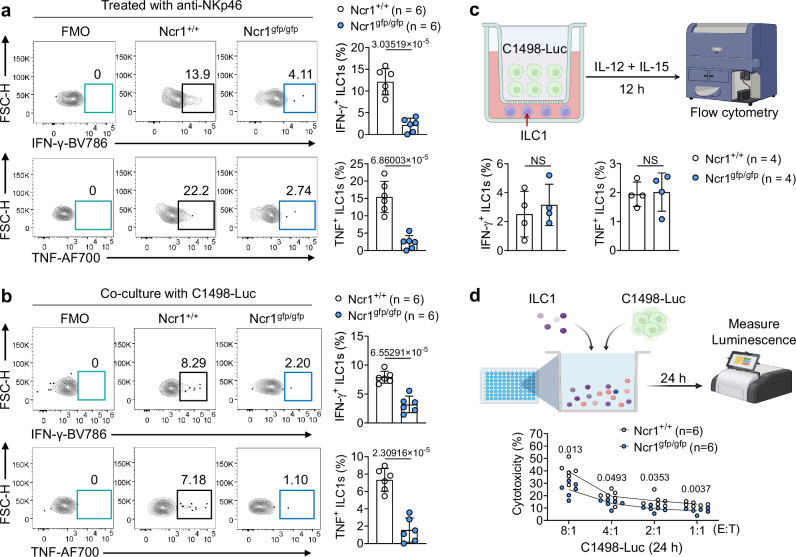
Engagement of NKp46 expressed on ILC1s augments their effector function. **a** *Ncr1*^*+/+*^ ILC1s and *Ncr1*^*gfp/gfp*^ ILC1s sorted from the liver of *Ncr1*^*+/+*^ and *Ncr1*^*gfp/gfp*^ mice, respectively, were cultured with or without anti-NKp46 antibody (5 μg/ml) for 12 h. Representative flow cytometry (left) and statistics (right) of the IFN-γ^+^ (top) and TNF^+^ (bottom) of ILC1s (*n* = 6). **b** *Ncr1*^*+/+*^ ILC1s and *Ncr1*^*gfp/gfp*^ ILC1s sorted from the liver of *Ncr1*^*+/+*^ and *Ncr1*^*gfp/gfp*^ mice, respectively, were cultured with or without C1498 AML cells expressing luciferase (C1498-Luc) for 12 h. Representative flow cytometry (left) and statistics (right) of the IFN-γ^+^ (top) and TNF^+^ (bottom) of ILC1s (*n* = 6). **c** *Ncr1*^*+/+*^ ILC1s and *Ncr1*^*gfp/gfp*^ ILC1s sorted from the liver of *Ncr1*^*+/+*^ and *Ncr1*^*gfp/gfp*^ mice, respectively, were cultured with C1498-Luc AML cells for 12 h. ILC1s were loaded into the bottom chambers of a 96-well Transwell plate. The top wells of the plate were loaded with C1498-Luc AML cells in the presence of IL-12 plus IL-15 cytokines. Statistics of the IFN-γ^+^ (left) and TNF^+^ (right) of ILC1s (*n* = 4). Created in BioRender. Ma, R. (2024) https://BioRender.com/a44t133. **d** *Ncr1*^*+/+*^ ILC1s and *Ncr1*^*gfp/gfp*^ ILC1s sorted from the liver of *Ncr1*^*+/+*^ and *Ncr1*^*gfp/gfp*^ mice, respectively, were cultured at the indicated ratios with C1498-Luc AML cells for 24 h. Luciferase activity in the wells with tumor cells was measured with a luminescence microplate reader (*n* = 6). Created in BioRender. Ma, R. (2024) https://BioRender.com/n74x942. Data were presented as means ± s.d. *P* values were calculated by either an unpaired Student’s *t*-test (**a**–**c**) or two-way ANOVA models with adjustments (**d**). NS not significant. Source data are provided as a Source Data file.

An essential effector function of ILC1s is to rapidly produce IFN-γ and TNF in response to inflammatory cytokines^3^. To determine whether activation of the NKp46 pathway is involved, we sorted ILC1s from mouse liver and cultured them with anti-NKp46 antibodies in the presence of IL-12 and IL-15 for 12 h. Our data showed that *Ncr1*^*+/+*^ ILC1s produced higher levels of IFN-γ and TNF compared to *Ncr1*^*gfp/gfp*^ ILC1s (Fig. 4a). Similar results were obtained when ILC1s were cocultured with the AML cell line expressing luciferase (C1498-Luc) in the presence of IL-12 and IL-15 for 12 h (Fig. 4b). However, when ILC1s were separated from C1498-Luc in a transwell system, the IFN-γ and TNF production was comparable between *Ncr1*^*+/+*^ ILC1s and *Ncr1*^*gfp/gfp*^ ILC1s (Fig. 4c). Our results suggest that ILC1s require direct contact with C1498 AML cells to produce IFN-γ and TNF. We also cocultured ILC1s with C1498-Luc for 24 h in the presence of IL-12 and IL-15 at various effector-to-target cell ratios (E: T) to examine whether NKp46 deletion affects ILC1 cytotoxicity. The luciferase luminescence-based assay showed that the *Ncr1*^*+/+*^ ILC1s were able to lyse C1498 AML cells more efficiently than their *Ncr1*^*gfp/gfp*^ counterparts (Fig. 4d). Collectively, these data reveal that ILC1s require NKp46 to produce IFN-γ and TNF, and to display cytotoxic effector against AML function in vitro.

### NKp46 is required for ILC1 proliferation in mice with AML and plays an important role in controlling AML in vivo

**Fig. 5:**
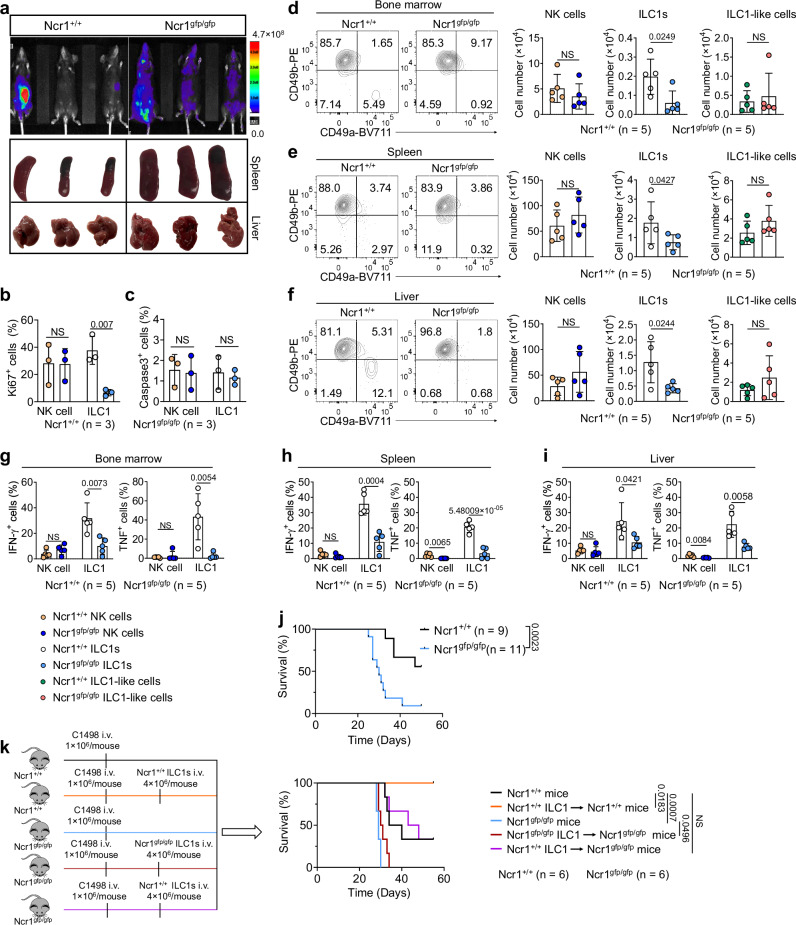
NKp46 is required for ILC1 proliferation in the tumor environment and plays an important role in controlling AML in vivo. *Ncr1*^*+/+*^ or *Ncr1*^*gfp/gfp*^ mice were i.v. injected with 1.0 × 10^6^ C1498-Luc AML cells. **a** Two weeks later, the tumor burden was assessed by bioluminescence imaging (*n* = 3). **b** Statistics of Ki67^+^ ILC1s and NK cells in the liver of *Ncr1*^*+/+*^ and *Ncr1*^*gfp/gfp*^ mice challenged with C1498-Luc AML cells (*n* = 3). **c** Statistics of caspase 3^+^ ILC1s and NK cells in the liver of *Ncr1*^*+/+*^ and *Ncr1*^*gfp/gfp*^ mice challenged with C1498-Luc AML cells (*n* = 3). **d**–**f** *Ncr1*^*+/+*^ or *Ncr1*^*gfp/gfp*^ mice were i.v. injected with 1.0 × 10^6^ C1498-Luc AML cells. Representative flow cytometry dot plots (left) and statistics (right) of the absolute number of NK cells, ILC1s, and ILC1-like cells in the bone marrow (**d**), spleen (**e**), and liver (**f**) of *Ncr1*^*+/+*^ and *Ncr1*^*gfp/gfp*^ ILC1s (*n* = 5). **g**–**i** Statistics of IFN-γ^+^ (left) and TNF^+^ (right) NK cells and ILC1s in the bone marrow (**g**), spleen (**h**), and liver (**i**) (*n* = 5). **j** Survival of the *Ncr1*^*+/+*^ and *Ncr1*^*gfp/gfp*^ mice injected with 1.0 × 10^6^ C1498-Luc AML cells (*Ncr1*^*+/+*^*:* *n* = 9; *Ncr1*^*gfp/gfp*^: *n* = 11). **k** *Ncr1*^*+/+*^ or *Ncr1*^*gfp/gfp*^ mice were i.v. co-injected with 1.0 × 10^6^ C1498-Luc AML cells and 4.0 × 10^6^ ILC1s isolated and expanded from *Ncr1*^*+/+*^ or *Ncr1*^*gfp/gfp*^ mice. Survival of the mice injected with C1498-Luc AML cells (*n* = 6). Survival data were analyzed by Kaplan–Meier survival analysis and log-rank test. Data were presented as means ± s.d. *P* values were calculated by an unpaired two-tailed Student’s *t*-test (**b**–**i**), one-tailed Student’s *t*-test (**e**: ILC1 panel), or log-rank test (**j**, **k**). NS not significant. Source data are provided as a Source Data file.

To investigate the functional relevance of NKp46 in vivo, we, i.v. injected C1498-Luc AML cells into *Ncr1*^*+/+*^ or *Ncr1*^*gfp/gfp*^ mice. After 2 weeks, we measured tumor burden with bioluminescence imaging. The *Ncr1*^*gfp/gfp*^ mice exhibited significantly more tumor growth and their spleens, but not their livers, were enlarged when compared to the *Ncr1*^+/+^ mice (Fig. 5a). These findings suggest that the absence of NKp46 led to accelerated tumor growth. Furthermore, we found that the percentage of Ki67^+^ ILC1s in the liver was significantly lower in the *Ncr1*^*gfp/gfp*^ mice compared to the *Ncr1*^*+/+*^ mice after the animals were challenged with AML cells, suggesting that NKp46 plays a role in ILC1 proliferation in response to AML (Fig. 5b). In contrast to ILC1s, the percentage of Ki67^+^ NK cells in the liver of *Ncr1*^*gfp/gfp*^ mice was not significantly different, when compared to *Ncr1*^*+/+*^ mice (Fig. 5b). We also found that apoptosis between the *Ncr1*^*+/+*^ and *Ncr1*^*gfp/gfp*^ ILC1s or between *Ncr1*^*+/+*^ and *Ncr1*^*gfp/gfp*^ NK cells was comparable (Fig. 5c). Thus, NKp46 does not appear to be necessary for regulating NK cell proliferation, nor does it significantly impact apoptosis in either ILC1s or NK cells within the C1498-Luc AML model. In follow-up experiments using the same AML mouse model, we observed that compared to wild-type (*Ncr1*^*+/+*^) animals, the number of ILC1s (CD49b^−^CD49a^+^) was lower in the bone marrow, spleen, and liver of *Ncr1*^*gfp/gfp*^ mice (Fig. 5d–f). These results contrast with the stable numbers of ILC1-like cells (CD49b^+^CD49a^+^) and NK cells (CD49b^+^CD49a^−^) that were unchanged across both genotypes (Fig. 5d–f). Additionally, the production of IFN-γ and TNF by ILC1s and ILC1-like cells was lower in the bone marrow, spleen, and liver of *Ncr1*^*gfp/gfp*^ mice transplanted with C1498-Luc AML cells, compared to *Ncr1*^*+/+*^ mice (Fig. 5g–i and Supplementary Fig. 6a–d). While the production of TNF also decreased in the spleen and liver of *Ncr1*^*gfp/gfp*^ NK cells compared to *Ncr1*^*+/+*^ NK cells, the extent of the decrease in TNF production was much more substantial in ILC1s compared to NK cells. In the bone marrow, we observed no significant changes in TNF production by NK cells, but there were differences in ILC1 IFN-γ and TNF production between *Ncr1*^*+/+*^ and *Ncr1*^*gfp/gfp*^ mice (Fig. 5g–i and Supplementary Fig. 6a, b). Finally, when implanted with C1498-Luc AML, the *Ncr1*^*gfp/gfp*^ mice exhibited significantly shorter survival compared to *Ncr1*^*+/+*^ mice (Fig. 5j), suggesting NKp46 plays an important role in controlling AML. This finding aligns with prior research indicating that patients with AML exhibiting high NKp46 expression at diagnosis tend to have better overall survival than those with low NKp46 expression^44^.

We next proceeded to perform adoptive transfer experiments using ILC1s. Injection of additional *Ncr1*^*+/+*^ ILC1s into *Ncr1*^*+/+*^ mice that were transplanted with C1498-Luc AML cells prolonged survival compared to control mice who were not provided with additional *Ncr1*^*+/+*^ ILC1 cells (Fig. 5k), supporting the notion that ILC1s play a role in anti-AML responses in vivo. We also injected equal numbers of *Ncr1*^*+/+*^ ILC1s and *Ncr1*^*gfp/gfp*^ ILC1s separately into *Ncr1*^*gfp/gfp*^ mice that had been transplanted with C1498-Luc AML cells to rescue the dramatic decrease in ILC1s observed in *Ncr1*^*gfp/gfp*^ mice, with no adoptive transfer of ILC1s used as a control. We observed that the injection of *Ncr1*^*+/+*^ ILC1s prolonged the survival of *Ncr1*^*gfp/gfp*^ mice with AML, compared to *Ncr1*^*gfp/gfp*^ mice that did not receive *Ncr1*^*+/+*^ ILC1 injections or were injected with *Ncr1*^*gfp/gfp*^ ILC1s (Fig. 5k). Collectively, our data demonstrate that (1) in our mouse model of AML, NKp46 deletion results in impaired ILC1 proliferation, reduced production of IFN-γ and TNF by ILC1s, and decreased survival of mice with AML; (2) injection of sufficient *Ncr1*^*+/+*^ ILC1s can prolong the survival of *Ncr1*^*gfp/gfp*^ mice with AML.

### Anti-NKp46 antibody and IL-7 have additive effects on enhancing NF-κB activity and IL-2Rα expression in ILC1s but not in NK cells

**Fig. 6:**
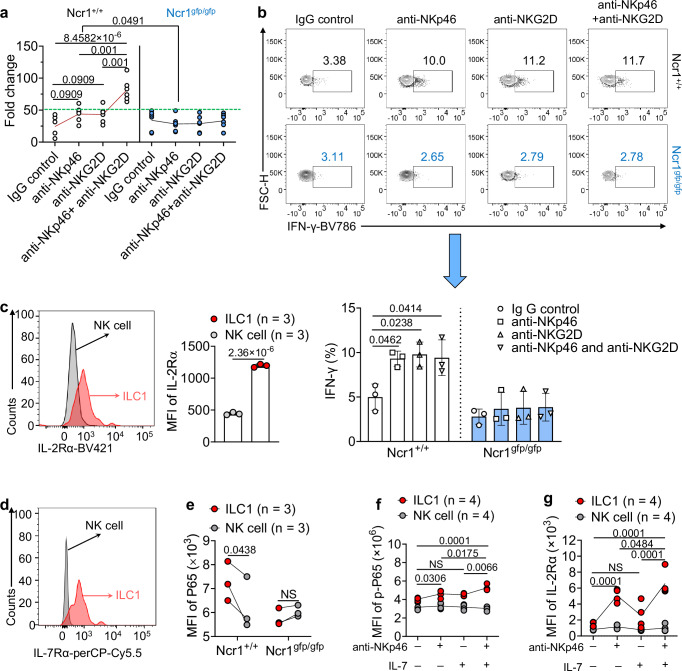
Collaboration between anti-NKp46 and IL-7 enhances NF-κB activity and IL-2Rα expression in ILC1s but not in NK cells. **a** NK cells were treated with or without anti-NKp46 antibody (5 μg/ml) in the presence or absence of NKG2D (5 μg/ml) for 7 days. The fold change of NK cell quantity was measured (*n* = 6). **b** NK cells were treated with or without anti-NKp46 antibody (5 μg/ml) in the presence or absence of NKG2D (5 μg/ml) for 4 h. Representative flow cytometry dot plots (top) and statistics (bottom) of the production of IFN-γ (*n* = 3). **c** Representative histogram (left) and quantification of IL-2Rα expression (right) on NK cells and ILC1s from *Ncr1*^*+/+*^ mice (*n* = 3). **d** Representative histogram of IL-7Rα expression on NK cells and ILC1s from *Ncr1*^*+/+*^ mice. **e** Statistics of P65 MFI on *Ncr1*^*+/+*^ ILC1s and NK cells or *Ncr1*^*gfp/gfp*^ ILC1s and NK cells (*n* = 3). **f** ILC1s or NK cells isolated from the liver of mice were treated with or without anti-NKp46 antibody (5 μg/ml) in the presence or absence of IL-7 (100 ng/ml) for 24 h. Statistics of phosphorylated P65 MFI (*n* = 4). **g** ILC1s or NK cells isolated from the liver of mice were treated with or without anti-NKp46 antibody (5 μg/ml) in the presence or absence of IL-7 (100 ng/ml) for 24 h. Statistics of IL-2Rα expression (*n* = 4). Data were shown as means ± s.d. *P* values were calculated by two-tailed Student’s *t-*test (**a**: *Ncr1*^*+/+*^ + anti-NKp46 vs *Ncr1*^*gfp/gfp*^ + anti-NKp46; **c**), one-tailed Student’s *t*-test (**e**), or one-way ANOVA (**a**, **b**) models with adjustments or two-way ANOVA with adjustments (**f**, **g**). NS not significant. Source data are provided as a Source Data file.

Next, we sought to determine the effect of engaging NKp46 signaling on ILC1s and NK cells. We observed a slightly but not significantly increased proliferation of liver *Ncr1*^*+/+*^ NK cells (Lin^−^NK1.1^+^NKp46^+^CD49b^*+*^CD49a^−^) when cultured with either an anti-NKp46 antibody or an anti-NKG2D antibody; however, a significant increase in proliferation of *Ncr1*^*+/+*^ NK cells occurred when both antibodies were used in combination, compared to cultures treated with either single antibody or without any antibody treatment (Fig. 6a). In contrast, the anti-NKp46 antibody and the anti-NKG2D antibody, either independently or in combination, activated *Ncr1*^*+/+*^ NK cells to produce IFN-γ (Fig. 6b). These results are consistent with previous reports indicating increased IFN-γ production in mouse NK cells activated by anti-NKp46 antibody alone^45^. Expectedly, the activation was ablated when *Ncr1*^*+/+*^ NK cells were replaced by *Ncr1*^*gfp/gfp*^ NK cells (Fig. 6b). However, combining anti-NKp46 antibody with anti-NKG2D antibody did not further augment the production of IFN-γ in *Ncr1*^*+/+*^ NK cells beyond levels achieved with either antibody alone, though the production was still higher than that of IgG control (Fig. 6b). Collectively, these results demonstrate that activation via NKp46 combined with activation via NKG2D enhances the proliferation of NK cells more effectively than either acting alone. However, this combination effect does not correspond to an increased production of IFN-γ in NK cells when compared to the activation of NKp46 or NKG2D alone. Additionally, these results confirm the functionality of the anti-NKp46 antibody that was used in our study. However, unlike its effects on ILC1s, we observed that the anti-NKp46 antibody alone failed to affect the expression of IL-2Rα on *Ncr1*^*+/+*^ NK cells (Supplementary Fig. 7a).

After culturing with IL-2 and IL-7, we observed that the expression of IL-2Rα was similar in both *Ncr1*^*+/+*^ NK cells and *Ncr1*^*gfp/gfp*^ NK cells (Supplementary Fig. 7b). Furthermore, the cytotoxicity against tumor cells exhibited by these NK cells was also comparable, both in vitro and in vivo (Supplementary Fig. 7c,d). This finding is consistent with a previous report^46^ that demonstrated *Ncr1*^*+/+*^ NK cells and *Ncr1*^*gfp/gfp*^ NK cells exhibit similar cytotoxicity against certain types of tumor cells (e.g., B10 and EL4). These data suggest that while the activation of NKp46 alone in liver NK cells can slightly enhance their proliferation, it is not essential for their cytotoxicity in vitro or in vivo and does not induce IL-2Rα expression in liver NK cells. In contrast, when comparing ILC1s and NK cells, we detected a significantly higher expression of IL-2Rα on liver ILC1s compared to liver NK cells (Fig. 6c). Additionally, we observed that liver NK cells did not express IL-7Rα, whereas ILC1s did (Fig. 6d), consistent with our previous report^47^. Furthermore, we found that the basal level of P65 expression was higher in *Ncr1*^*+/+*^ ILC1s compared to *Ncr1*^*+/+*^ NK cells (Fig. 6e). However, this difference was not found when comparing *Ncr1*^*gfp/gfp*^ ILC1s with *Ncr1*^*gfp/gfp*^ NK cells (Fig. 6e). These data provide an explanation as to why NF-κB signaling is more sensitive to activation by anti-NKp46 antibody in ILC1s compared to NK cells.

Given the critical role that IL-7 plays in the development of ILC1s, as opposed to its negligible impact on NK cells^10^, we examined whether IL-7 promotes anti-NKp46 antibody-induced phosphorylation of P65 and the expression of IL-2Rα in ILC1s. For this purpose, we treated ILC1s with anti-NKp46 antibody in the presence or absence of IL-7. As anticipated, the combination of anti-NKp46 antibody and IL-7 led to higher P65 phosphorylation and IL-2Rα expression in ILC1s compared to either single-agent treatment or the no-agent condition (Fig. 6f, g). While each single agent alone increased the phosphorylation of P65 and expression of IL-2Rα in ILC1s, this enhancement was not present in NK cells (Fig. 6f, g). These data suggest that IL-7 enhances NKp46-mediated NF-κB activation and IL-2Rα expression in ILC1s but not in NK cells.

### Human NKp46^+^ ILC1s are more proliferative and cytotoxic than their NKp46^−^ counterparts

**Fig. 7:**
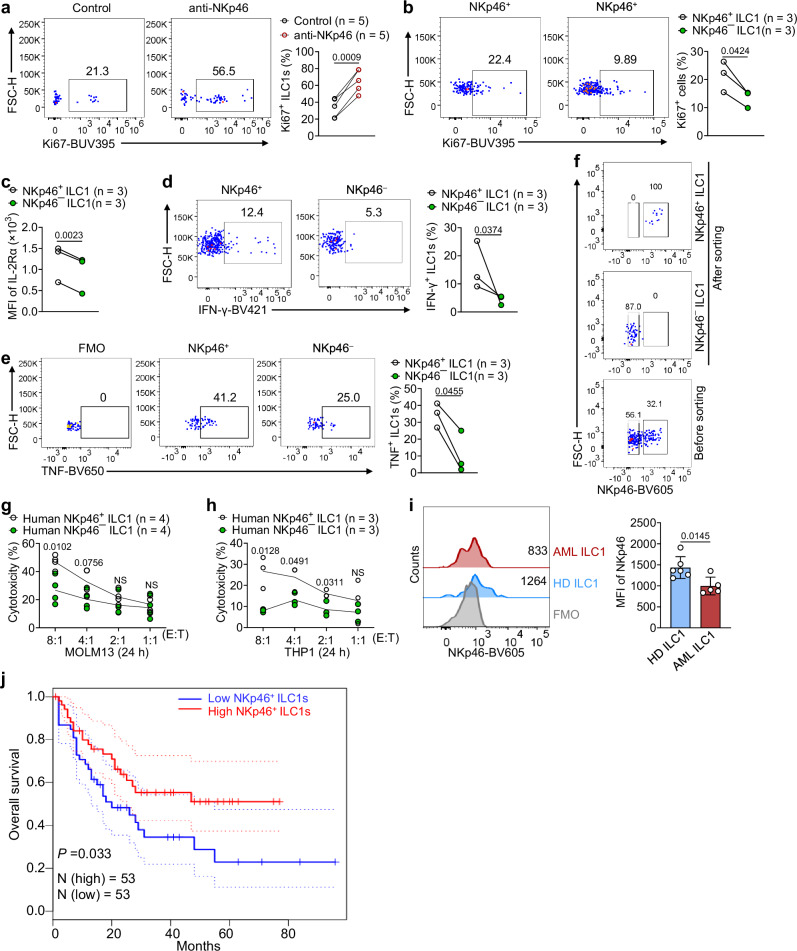
NKp46 is required for human ILC1 proliferation, cytokine production, and cytotoxicity against human AML. **a** Human ILC1s isolated from peripheral blood mononuclear cells (PBMCs) were treated with or without human anti-NKp46 antibodies (5 μg/ml) for 3 days. Representative flow cytometry plots of Ki67^+^ ILC1s are presented. Statistics of Ki67^+^ ILC1s (*n* = 5). **b** Representative flow cytometry plots (left) and statistics (right) of Ki67 expression in human NKp46^+^ and NKp46^*−*^ ILC1s (*n* = 3). **c** Statistics of IL-2Rα expression in human NKp46^+^ and NKp46^*−*^ ILC1s (*n* = 3). **d** Representative flow cytometry plots (left) and statistics (right) of IFN-γ production in human NKp46^+^ and NKp46^*− *^ILC1s (*n* = 3). **e** Representative flow cytometry plots (left) and statistics (right) of TNF production in human NKp46^+^ and NKp46^*− *^ILC1s (*n* = 3). **f** Gating strategy for sorting human NKp46^+^ and NKp46^*− *^ILC1s. **g**, **h** Human NKp46^+^ and NKp46^*− *^ILC1s sorted from human PBMCs were cultured at the indicated ratios with MOML13-Luc or THP-1-Luc for 24 h. Luciferase activity in the wells with tumor cells was measured with a luminescence microplate reader (**g**, *n* = 4; **h**, *n* = 3). **i** Histogram (left) and statistics analysis showing the expression of NKp46 on ILC1s isolated from the blood of healthy donors (HD ILC1s) or patients with AML (AML ILC1s) (*n* = 6 individuals in the healthy donor group, *n* = 5 individuals in the AML patient group). **j** Survival analyses based on ILC1-related signature genes (*IFNG*, *TNFA*, *TRAIL*, *RORC*, *NKp46*, *CD49A*, *CSF2*, and *LTA*) in the TCGA-LAML cohort (*n* = 106). Log-rank Mantel–Cox test. Data were presented as means ± s.d. *P* values were calculated by two-tailed Student’s *t*-test (**a**–**i**). A log transformation was used in (**d**). A Student’s *t*-test was conducted at different ratios of effector cells to target cells in panels (**g**, **h**). NS not significant. Source data are provided as a Source Data file.

In subsequent studies focusing on human cells, we investigated whether NKp46 is required for proliferation and cytotoxicity in human ILC1s. First, we isolated human ILC1s from peripheral blood mononuclear cells (PBMCs) (Supplementary Fig. 2c) and cocultured them with a human anti-NKp46 antibody prior to examining the expression of the proliferation marker Ki67. As expected, activating the NKp46 signaling pathway increased Ki67 expression in human ILC1s (Fig. 7a). We also observed that basal expression of Ki67 was lower in human NKp46^− ^ILC1s compared to NKp46^+^ ILC1s (Fig. 7b). Additionally, in line with our findings with murine *Ncr1*^*gfp/gfp*^ ILC1s, human NKp46^− ^ILC1s had lower IL-2Rα expression compared to NKp46^+^ ILC1s (Fig. 7c). These data suggest that NKp46 may also be required for the proliferation of human ILC1s and that NKp46 expression correlates with IL-2Rα expression on human ILC1s. Furthermore, when human ILC1s were cocultured with the human AML cell line MOLM13, the NKp46^+^ ILC1s produced more IFN-γ and TNF than their NKp46^− ^counterparts (Fig. 7d, e). Notably, while the cytokine production by these cells and the markers we used to define them suggest that they are NKp46^+^ ILC1s and NKp46^− ^ILC1s, we can not exclude the possibility that less cytokine production NKp46^− ^ILC1s could be because NKp46^− ^ILC1s are immature precursors of NKp46^+^ ILC1s. Indeed, we recently used NKp46 to define the maturation stages of murine NK cells^48^. To further assess the effect of NKp46 on the cytotoxicity of human ILC1s, we sorted NKp46^− ^and NKp46^+^ ILC1s from human PBMCs and cocultured them with MOLM13 AML at various effector and target cell ratios for 24 h. As anticipated, the human NKp46^− ^ILC1s were significantly less cytotoxic compared to NKp46^+^ ILC1s (Fig. 7f, g). We achieved similar results when we used ILC1s isolated from human PBMCs cocultured with the human AML cell line THP-1 (Fig. 7h). Taken together, these data indicate that, as in mice, human NKp46^+^ ILC1s are more proliferative and cytotoxic than their NKp46^−^ counterparts. Extending our investigation to patients with AML, we observed a significant decrease in NKp46 expression on ILC1s in the blood of patients with AML compared to healthy donors (Fig. 7i). We further employed ILC1-related markers (*IFNG*, *TNFA*, *TRAIL*, *NKp46*, *CD49A*, and *CSF2*) to analyze data from The Cancer Genome Atlas (TCGA)^49,50^. The analysis data revealed that AML patients with high NKp46^+^ ILC1s tended to experience longer overall survival compared to AML patients with low NKp46^+^ ILC1s (Fig. 7j). However, a limitation of this analysis is that *IFNG*, *TNFA*, *TRAIL*, *NKp46*, *CD49A*, and *CSF2* are not specific to ILC1s, and the separation of human ILC1s from NK cells based on markers remains imprecise. Collectively, our findings highlight the conserved role of NKp46 in regulating the proliferative and cytotoxic capacities of ILC1s across species and suggest its potential prognostic value in the context of AML.

## Discussion

We and others previously demonstrated that NKp46 plays an important role in the development and function of mouse ILC1s^29,30,51^. However, the precise mechanisms involved remain unclear. In this study, we describe a previously unknown association between the expression of NKp46 and certain cytokine receptors (e.g., IL-18R1, IL-7Rα, and IL-2Rα) on ILC1s. NKp46 expression induced the NF-κB signaling pathway and led to the expression of IL-2Rα, which in turn maintained ILC1 proliferation in vitro and in vivo. Our findings also revealed that the expression of NKp46 on ILC1s is critical for their interaction with AML cells and subsequent enhancement of NKp46-mediated cytotoxicity. Mice lacking NKp46 expression on ILC1s showed reduced regulation/control of AML in vivo.

It has been demonstrated that memory ILC1s undergo local proliferation in the liver and express high levels of IL-18R1, IL-7Rα, and IL-2Rα in the post-infected mouse cytomegalovirus (MCMV) model^15^. Analysis of the NKp46-deficient mouse revealed that ILC1s reduce the expression of their cytokine receptors (IL-18R1, IL-7Rα, and IL-2Rα) in a steady state, indicating that NKp46 may play an important role in the acquisition of phenotypic markers of memory ILC1 by controlling IL-18R1, IL-7Rα, and IL-2Rα expression. This possibility is also supported by the finding that IL-7Rα is a marker of memory ILC1s that is critical for their long-term homeostasis and IL-7Rα^+^ ILC1s acquire their memory through IL-7R signaling when they respond to haptens^52^.

NKp46 has been long recognized as a marker expressed by other ILC subsets in addition to NK cells^53,54^, and plays a critical role in regulating TRAIL surface expression^29,53^. However, its role in cell types other than NK cells has been obscure. We have previously reported that loss of NKp46 notably reduces the percentages and numbers of ILC1s in mice^30^, though the mechanism remained unclear. This report provides evidence that NKp46 deficiency does not affect the survival of ILC1s but impairs their proliferation by decreasing the expression of IL-2Rα, as demonstrated in both in vitro and in vivo experiments. This finding explains why both the percentages and numbers of ILC1s dramatically decreased in NKp46-deficient mice. Our study also suggests that modulating NKp46 signaling may facilitate the ex vivo expansion of ILC1s for future clinical applications. Although NK cells also express NKp46, we have not observed a similar regulation of IL-2Rα and the associated cell proliferation via NKp46 signaling in NK cells. Although the exact reason for this discrepancy between ILC1s and NK cells is unknown, we speculate that this may be related to higher basal levels of NF-κB P65 signaling and its IL-2Rα target in ILC1s in contrast to NK cells, as we demonstrated.

CD3ζ and FcξRγ are both downstream of NKp46. These immune adapters possess ITAM motifs that, when phosphorylated, activate the signaling proteins Syk and ZAP70 to boost cytotoxicity and/or cytokine production in NK cells^55^. However, in the current study, we demonstrated an alternative NKp46 signaling pathway in which engagement of NKp46 promotes NF-κB signaling; interestingly, this identified mechanism uniquely occurs in ILC1s but not in NK cells. In support of this, unlike ILC1s, we found NKp46 activation in vitro failed to increase NF-κB activity or IL-2Rα expression in NK cells. This does not exclude an activation role for NKp46 in NK cells, for which, unlike ILC1s, activation of a co-receptor may be required. This speculation is supported by the fact that NKp46 collaborated with other activating receptors, such as NKG2D, 2B4 (CD244), DNAM-1 (CD226), or CD2, to activate NK cells^56,57^.

The potent anti-cancer properties of NK cells in innate and adaptive immunity have been extensively reported, but only a few published studies have assessed how ILC1s combat tumorigenesis. A previous study reported that the blood and bone marrow of patients with AML had elevated percentages of ILC1s among total ILCs^58^, though absolute cell numbers and proportions in large populations such as lineage-negative cells were not provided. Moreover, the underlying mechanism by which ILC1s influence the development of AML remains unknown. We recently demonstrated that IFN-γ produced by ILC1s controls AML by promoting leukemia stem cell (LSC) apoptosis, inhibiting LSC differentiation into leukemia progenitor cells, and by favoring LSC differentiation into non-leukemia cells^47^. In the current study, through the use of NKp46-deficient mice in which the number of ILC1s is dramatically decreased, we demonstrated that tumor growth was accelerated and survival was shortened. This finding underscores the unique and essential contribution of NKp46^+^ ILC1s in the control of AML, in distinct contrast to that of NKp46^+^ NK cells. Indeed, a comparable role of NKp46 was not observed among NK cells, as animals showed no difference in survival regardless of whether they received injections of *Ncr1*^*+/+*^ or *Ncr1*^*gfp/gfp*^ NK cells into *Rag2*^*−/−*^*IL-2γc*^*−/−*^ mice challenged with an AML cell line. This is also supported by a report showing that *Ncr1*^*+/+*^ NK cells and *Ncr1*^*gfp/gfp*^ NK cells exhibit similar cytotoxicity against tumor cell lines (e.g., B10 and EL4)^46^. These results suggest NKp46 influences ILC1s instead of NK cells to control disease in the C1498 AML model. Therefore, expanding autologous ILC1s or normal allogeneic ILC1s ex vivo, using an anti-NKp46 antibody or ligands, might positively impact the survival of patients with AML. Expanded ILC1s could also be rationally combined with a Food and Drug Administration (FDA)-approved drug, such as a tyrosine kinase inhibitor, including FDA-approved midostaurin and gilteritinib.

In conclusion, our findings reveal a previously unknown functional role for NKp46: it regulates IFN-γ and TNF production, cytotoxicity, and the proliferation of ILC1s by activating the NF-κB signaling pathway and regulating IL-2Rα expression. These effects are important for anti-tumor activity, as exemplified in our model of AML.

## Methods

### Human samples

Peripheral blood (PB) samples were obtained from healthy donors (HDs) and acute myeloid leukemia (AML) patients at the City of Hope National Medical Center (COHNMC). Human specimens were collected from individuals with AML registered at the COHNMC who consented to an Institutional Review Board (IRB)-approved protocol (IRB 18067); healthy donor specimens were collected from individuals who consented to IRB 06229. Recruitment in this cohort was independent of sex, as any patient with newly diagnosed, relapsed, or refractory AML was eligible. All participants in this study were patients with relapsed or refractory acute myeloid leukemia. Information on race, ethnicity, or other socio-economical parameters was not collected for this cohort. Given the limited size of the cohort, we did not conduct any analyses stratified by sex. Patients who met the inclusion criteria outlined above were recruited for this study, so we do not anticipate any self-selection or other biases.

### Animals

Six- to eight-week-old male or female mice were used for all animal experiments. NKp46-deficient C57BL/6 mice—with *Ncr1* replaced with gfp (*Ncr1*^*gfp/gfp*^)—were previously described^59^. C57BL/6 mice (*Ncr1*^*+/+*^) and *Rag2*^*−/−*^*γc*^*−/−*^ mice (C57BL/6 background) were purchased from The Jackson Laboratory. Mice were housed in the City of Hope Animal Facility with a 12-h light/12-h dark cycle and temperatures of ~18–23 °C with 40–60% air humidity. Mouse care and experimental procedures were performed in accordance with federal guidelines and protocols approved by the Institutional Animal Care and Use Committee (IACUC) at City of Hope under protocol numbers 18108 and 20003. Tumor-bearing mice were monitored twice per week and at more frequent intervals depending on the status of the mice. Mice exhibiting evidence of distress, discomfort, pain, lethargy, inability to properly groom, or inability to obtain food and/or water were killed immediately via CO_2_ inhalation. Tumor-bearing mice with 20% weight loss from the age-matched controls without receiving tumor cell inoculation were killed.

### Cells and cell culture

Mouse ILC1s were cultured in RPMI 1640 (Thermo Fisher Scientific, USA) with 10% FBS, penicillin (100 U/ml), streptomycin (100 mg/ml), IL-2 (1000 IU/ml), and IL-7 (100 ng/ml). Mouse NK cells were cultured in RPMI 1640 with 10% FBS, penicillin (100 U/ml), streptomycin (100 mg/mL), and IL-2 (1000 IU/ml).

The mouse AML cell line C1498 (Cat# TIB-49) and the human AML cell line THP-1 (Cat# TIB-202) were purchased from the American Type Culture Collection (ATCC). The human AML cell line MOLM13 (Cat# ACC-554) was purchased from the Leibniz Institute DSMZ - German Collection of Microorganisms and Cell Cultures GmbH. All cell lines were cultured in RPMI 1640 with 10% FBS, penicillin (100 U/ml), and streptomycin (100 mg/ml). All cell lines were routinely tested for the absence of Mycoplasma using the MycoAlert Plus Mycoplasma Detection Kit from Lonza (Cat# M7006). Cultures were incubated at 37 °C in a humidified atmosphere of 5% CO_2_. All cytokines were from PeproTech. Penicillin and streptomycin were from Thermo Fisher Scientific.

### Flow cytometry

ILC1s from mice were identified using live/dead cell viability dyes and the following monoclonal antibodies: lineage (PE-Cy7-conjugated anti-CD3, BD Biosciences Cat# 552774, Clone: 145-2C11; PE-Cy7-conjugated anti-CD19: BD Biosciences, Cat# 552854, Clone: 1D3), NK1.1 (BV510-conjugated anti-NK1.1, BD Biosciences Cat# 563096, Clone: PK136), NKp46 (AF647-conjugated anti-NKp46, BD Biosciences, Cat# 560755, Clone: 29A1.4), CD49b (BUV395-conjugated anti-CD49b, BD Biosciences, Cat# 740250, Clone: HMα2 or PE-conjugated anti-CD49b: BD Biosciences, Cat# 553858, Clone: DX5), and CD49a (BV711-conjugated anti-CD49a, BD Biosciences, Cat# 564863, Clone: Ha31/8). *Ncr1*^*+/+*^ mouse ILC1s were identified by Lin^***−***^NK1.1^+^ NKp46^+^ CD49b^***−***^CD49a^+^, and *Ncr1*^*gfp/gfp*^ mouse ILC1s were gated by Lin^***−***^NK1.1^+^GFP^+^CD49b^***−***^CD49a^+^. In some experiments, ILC1s were identified by Lin^***−***^NK1.1^+^CD200R^+^CD49b^***−***^CD49a^+^ or Lin^***−***^NK1.1^+^CD49b^***−***^CD49a^+^. Intracellular staining for Ki67 and Caspase 3 was performed using a Fix/Perm kit (eBiosciences, Cat# 88-8824-00) followed by staining with Ki67 (BUV395-conjugated anti-Ki67, BD Biosciences, Cat# 564071, Clone: B56) or caspase 3 (BV421-conjugated anti-Caspase 3, BD Biosciences Cat# 570786, Clone: C92-605.rMAb) antibodies. Intracellular staining for GzmA (PerCP-e710-conjugated anti-GzmA, Thermo Fisher Cat# 46-5831-82, Clone: GzA-3G8.5), GzmB (PE-conjugated anti-GzmB, BioLegend Cat# 372208, Clone: QA16A02), and GzmC (BV421-conjugated GzmC, BD Biosciences Cat# 569861, Clone: SFC1D8.rMAb) antibodies was performed using a Cytofix/Cytoperm™ Fixation/Permeabilization Kit (BD Biosciences, Cat# 554714). In some experiments, caspase 3 was measured with the Active Caspase 3 Apoptosis Kit (BD Biosciences, Cat# 565521) according to the manufacturer’s instructions. Annexin V dead cell staining was conducted according to the instructions of the Annexin V Apoptosis Detection Kit (BioLgend, Cat# 640947). For IL-2Rα, IL-7Rα, and IL-18R1 staining, cells were collected from the liver of mice, and then washed three times with DPBS containing 1% FBS, followed by staining with lineage (PE-Cy7-conjugated anti-CD3 and anti-CD19), NK1.1 (BV510-conjugated anti-NK1.1), NKp46 (AF647-conjugated anti-NKp46), CD49b (BUV395- or PE-conjugated anti-CD49b), CD49a (BV711-conjugated anti-CD49a), IL-2Rα (BV605-conjugated anti-IL-2Rα, BioLegend Cat# 102036, Clone: PC61), IL-7Rα (PE-Cy5.5-conjugated anti-IL-7Rα, BioLegend, Cat# 121114, Clone: SB/199 or BV421-conjugated anti-IL-7Rα, BioLegend, Cat# 121127, Clone: SB/199), and IL-18R1 (PerCP-eFluor™ 710-conjugated anti-IL-18R1, Thermo Fisher, Cat #46-5183-82, Clone: P3TUNYA).

To examine intracellular cytokine production, ILC1s were cocultured with or without anti-NKp46 antibody (Thermo Fisher, Cat# 16-3351-81, Clone: 29A1.4) or tumor cells in the presence of mouse IL-12 (10 ng/ml) and mouse IL-15 (100 ng/ml) for 4 or 12 h. For all of the above stimulation assays, BD GolgiPlug™ was added to the cultures 4 h before cells were collected. Then cells were harvested, washed, and stained for surface molecules and intracellular IFN-γ and TNF. Intracellular staining for IFN-γ or TNF was performed using a BD Cytofix/Cytoperm™ Fixation/Permeabilization Kit, followed by staining with an IFN-γ antibody (BV786-conjugated anti-mouse IFN-γ, BD Biosciences, Cat# 563773, Clone: XMG1.2/BV421-conjugated anti-human IFN-γ, BioLegend, Cat# 506538, Clone: B27) or a TNF antibody (AF700-conjugated anti-mouse TNF, BioLegend, Cat# 506338, Clone: MP6-XT22/BV650-conjugated anti-human TNF, BioLegend, Cat# 502938, Clone: MAb11), respectively. All analyses were performed on a Fortessa X-20 flow cytometer (BD Biosciences), and sorting was performed using a BD FACSAria^TM^ Fusion (BD Biosciences).

For STAT3, STAT5, or P65 phosphorylation analysis, freshly isolated mouse liver cells were stained with Lineage, NK1.1, CD49b, CD49a, and/or NKp46 antibodies. Thirty minutes later, cells were washed three times with DPBS containing 1% FBS, and then were incubated for 15, 30, or 60 min at 37 °C as indicated with either IL-2 (1000 IU/ml), IL-7 (100 ng/ml), IL-18 (10 ng/ml), or anti-NKp46 antibody (5 μg/ml). Cells were directly fixed with BD Phosflow™ Perm Buffer I (BD Biosciences, Cat# 557870), followed by permeabilization using a BD Cytofix/Cytoperm™ Fixation/Permeabilization Kit according to the manufacturer’s instructions. They were then stained with p-STAT3 (BV421-conjugated anti-p-STAT3, BioLegend, Cat# 651010, Clone: 13A3-1), p-STAT5 (PE-conjugated anti-p-STAT5, Cell Signaling Technology, Cat# 14603, Clone: D47E7), or p-P65 (PE-conjugated anti-p-P65, Cell Signaling Technology, Cat# 653004, Clone: 14G10A21) antibodies in flow cytometry buffer. All cytokines were from Peprotech or the National Institutes of Health. All analyses were performed on a Fortessa X-20 flow cytometer (BD Biosciences), and sorting was performed using a BD FACSAria^TM^ Fusion.

### In vitro stimulation experiments

Flat-bottom plates were coated with 5 μg/ml isotype (Thermo Fisher, Cat # 16-4321-82, Clone: eBR2a) or 5 μg/ml anti-NKp46 antibody (Thermo Fisher, Cat# 16-3351-81, Clone: 29A1.4) in DPBS for three hours at 37 °C or overnight at 4 °C. The plates were washed twice with DPBS plus 1% FBS. FACS-sorted mouse ILC1s or NK cells were resuspended in RPMI 1640 plus 10% FBS for the indicated time. The stimulation was performed similarly to the human NK cell experiments.

### ILC1 and NK cell expansion in vitro

Mouse ILC1s or NK cells were sorted from the liver of mice, and then were cultured in RPMI 1640 with 10% FBS, penicillin (100 U/ml), streptomycin (100 mg/ml), IL−2 (1000 IU/ml), and/or IL-7 (100 ng/ml) for indicated times at 37 °C. The cells were counted using a hemacytometer. A trypan blue solution (Thermo Fisher, Cat# 15250061) was used to exclude dead cells.

### In vivo labeling of mouse cells with BrdU

A 10 mg/ml solution of bromodeoxyuridine (BrdU) in sterile 1× DPBS was provided for in vivo use. Six- to eight-week-old male or female *Ncr1*^*+/+*^ or *Ncr1*^*gfp/gfp*^ mice were intraperitoneally (i.p.) injected with 200 µl of BrdU solution. Sixteen hours later, the mice were euthanized. The ILC1s in the liver of mice were stained with a BrdU antibody (BV510-conjugated anti-BrdU, Cat# 563445, Clone: 3D4). The BrdU^+^ ILC1s were detected using flow cytometry.

### ILC1 transplantation

*Ncr1*^*+/+*^ (GFP-negative) or *Ncr1*^*gfp/gfp*^ (GFP-positive) ILC1s were sorted from the liver of six- to eight-week-old male or female mice, labeled with Cell Trace Violet (CTV; Thermo Fisher, Cat# C34571), mixed at a 1:1 ratio, and then intravenously (i.v.) injected into six- to eight-week-old male or female *Rag*^*–/–*^*γc*^*–/–*^mice. One week later, the mice were euthanized. The ILC1s in the liver of mice were stained with lineage, NK1.1, CD200R (APC-conjugated anti-CD200R, Thermo Fisher Cat# 17-5201-82, Clone: OX110), CD49b, and CD49a antibodies. Lin^**−**^NK1.1^+^CD200R^+^CD49b^**−**^CD49a^+^ were used to identify ILC1s. The GFP^+^ ILC1s and GFP^**−**^ ILC1s were detected using flow cytometry.

### In vitro killing assay

Various numbers of FACS-sorted liver ILC1s or NK cells were cultured at the indicated ratios with 300 C1498-Luc cells. After 24 h, 100 μl of the mixture was transferred to a 96-well white luminometer plate. Next, 10 μl of the substrate (Promega, Cat# E2510) was added, and luminescence relative light unit (RLU) values were immediately determined. The results are reported as percent killing based on luciferase activity in the wells with tumor cells but no ILC1s [% killing = 100 – [(RLU) from well with effector and target cell co-culture)/(RLU from wells with target cells) × 100)]^60^.

### AML model experiment

Six- to eight-week-old male or female *Ncr1*^*+/+*^ or *Ncr1*^*gfp/gfp*^ mice were i.v. injected with 1.0 × 10^6^ C1498-Luc AML cells, followed by i.v. injection of 4.0 × 10^6^ ILC1s or NK cells. Tumor burden was assessed via in vivo bioluminescence measurements using the IVIS Imaging System at the City of Hope Imaging Center. For luciferase detection imaging, 200 μl of 15 mg/ml D-luciferin (Biosynth, Cat# L-8220) in DPBS was injected i.p. before imaging. Three weeks later, the mice were euthanized, and the number of ILC1s and NK cells, the production of IFN-γ and TNF in the bone marrow, spleen, and liver of ILC1s and NK cells, the proliferation marker Ki67, and the apoptosis marker caspase 3, were analyzed by flow cytometry. Mouse survival was monitored every day.

### RNA sequencing (RNA-seq)

*Ncr1*^*+/+*^ or *Ncr1*^*gfp/gfp*^ ILC1s were sorted from the liver of six- to eight-week-old male or female mice using BD FACSAria^TM^ Fusion. Total RNA was isolated from ILC1s using a miRNeasy Mini Kit (QIAGEN). PolyA RNA-seq was performed in the Integrative Genomics Core of the City of Hope National Medical Center. A SMART-Seq® Ultra Low Input RNA Kit for Sequencing–v4 (Takara Bio) was used to obtain double-stranded cDNA from each sample, with a total input of 2 ng RNA. The resulting cDNA was sheared using a Covaris LE220 sonicator. The sheared DNA was used to prepare a sequencing library, using a KAPA HyperPrep Kit. The final libraries were quantified using the Qubit Assay Kit (Thermo Fisher Scientific) and Bioanalyzer (Agilent). Sequencing was performed using the single-read mode of 51 cycles of read 1 and 7 cycles of the index read with V4 reagents on a Hiseq 2500 system (Illumina). Real-time analysis (RTA) 2.2.38 software was used for image analysis and base calling. RNA-seq reads were trimmed to remove sequencing adapters, using Trimmomatic and polyA tails and using Fast Analysis of Sequencing Tab Princlip (FASTP)^61^. The processed reads were mapped back to the mouse genome (mm10), using STAR software (v. 020201)^62^. The HTSeq software (v.0.6.0) was used to generate the count matrix, with default parameters^63^. Differential expression analysis was conducted by adjusting read counts to normalized expression values, using the trimmed mean of M values (TMM) normalization method in R^64^. Briefly, for cell-type comparison, general linear models were applied to identify differentially expressed genes (DEGs) between two specific cell types, using TMM normalization expression level as the dependent variable and cell type as the independent variable. Genes with a Franklin Delano Roosevelt (FDR) adjusted *P* value < 0.05 and a fold change (FC) > 1.5 or < 0.7 were considered significant up- or down-regulated genes, respectively. Pathway analysis was conducted with the gene set enrichment analysis (GSEA) Preranked algorithm, using the GSEA Desktop program in Java^65,66^. For gene set enrichment analysis, we used a knowledge-based approach for interpreting the genome that required a ranked list of whole genes according to their log2 fold change and *p* values.

### Statistical analysis

For continuous endpoints, we used Student’s *t*-test or paired *t-*test to compare two independent or paired conditions, and one-way or two-way ANOVA models to compare three or more independent conditions. For repeated measures (e.g., measures over time or matched groups), linear mixed models were used to account for the variance and covariance structure. For mouse survival data, the Kaplan–Meier method and log-rank test were used to estimate survival functions and to compare different groups. *P* values were adjusted for multiple comparisons by Holm’s procedure when necessary. A *P* value of 0.05 or less is considered statistically significant. No statistical methods were used to predetermine sample sizes, but our sample sizes are similar to those reported in previous publications^47^. No data were excluded from the analyses. Experimenters were blinded to observe the survival of mice. Otherwise, blinding was not performed, such as during in vitro experiments, where experimenters were required to know the conditions of each well. Data distribution was assumed to be normal, but this was not formally tested. Sequencing reads were trimmed from sequencing adapters using Trimmomatic^67^ and polyA tails using FASTP^61^, and then mapped back to the mouse genome (mm10) using STAR (v. 020201)^62^. The gene-level count table was created by a high-throughput sequence (HTSeq v.0.6.0)^63^ and normalized by the trimmed mean of M values (TMM)^64^ method. General linear models based on negative binomial distributions (R package “EdgeR”) were used to compare gene expression levels between two specific cell types. Genes with a false discovery rate (FDR)-adjusted *P* value < 0.05 and a fold change (FC) > 1.5 (upregulated) or < 0.7 (downregulated) were considered to be DEG. Pathway and gene set enrichment analyses were performed using the GSEA^65,66^ program, which runs the GSEAPreranked algorithm on a ranked list of genes. Data were presented as means ± s.d. Prism software 9.3.1 (GraphPad, CA, USA) and SAS v.9.4 (SAS Institute. NC, USA) were used to perform statistical analyses.

### Reporting summary

Further information on research design is available in the Nature Portfolio Reporting Summary linked to this article.

## Supplementary information

Supplementary Information

Reporting Summary

## Source data

Source Data

## Data Availability

Source data are provided with this paper. The RNA-seq data used in this study are accessible in GEO under accession code GSE283199. The remaining data are available within the article, Supplementary Information or Source Data file. Source data are provided with this paper.
