# Supplementary material for: NKp46 enhances type 1 innate lymphoid cell proliferation and function and anti-acute myeloid leukemia activity
Source: Nat Commun. 2025 Jan 24;16:989. doi: 10.1038/s41467-025-55923-w (PMC11760942; doi:10.1038/s41467-025-55923-w)
Supplement: Supplementary file 1 — Supplementary Information [file 41467_2025_55923_MOESM1_ESM.pdf]

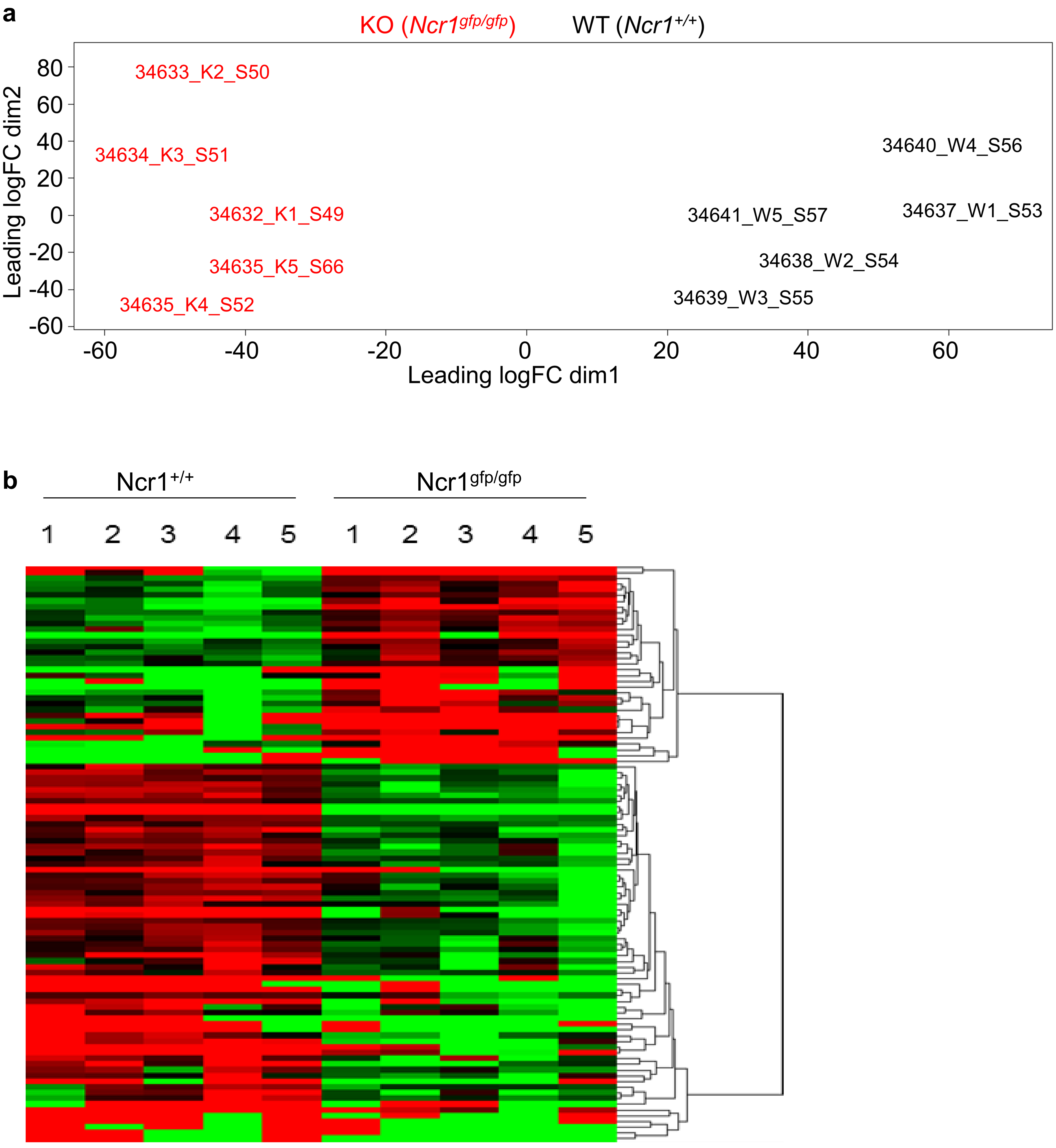
a
KO (Ncr1gfp/gfp)
WT (Ncr1+/+)
34633_K2_S50
34634_K3_S51
34632_K1_S49
34635_K5_S66
34635_K4_S52
34640_W4_S56
34641_W5_S57
34637_W1_S53
34638_W2_S54
34639_W3_S55
Leading logFC dim2
Leading logFC dim1
80
60
40
20
0
-20
-40
-60
-60
-40
-20
0
20
40
60
b
Ncr1+/+
Ncr1gfp/gfp
1 2 3 4 5
1 2 3 4 5

Supplementary Fig. 1 RNA-seq identifies upregulated and downregulated genes in *Ncr1*$^{+/+}$ ILC1s and *Ncr1*$^{gfp/gfp}$ ILC1s. *Ncr1*$^{+/+}$ ILC1s and *Ncr1*$^{gfp/gfp}$ ILC1s were sorted for RNA-Seq using FACS from the liver of *Ncr1*$^{+/+}$ and *Ncr1*$^{gfp/gfp}$ mice, respectively. (a) The multidimensional scaling plot shows the separation of *Ncr1*$^{+/+}$ ILC1s and *Ncr1*$^{gfp/gfp}$ ILC1s samples. The names with letters (W for WT and K for knock-out) and numbers are displayed. (b) Heat map showing differential expression of RNA of 101 genes (n = 5). Genes with a false discovery rate (FDR)-adjusted *P*-value < 0.05 and a fold change (FC) > 1.5 or < 0.7 were considered as significant upregulated and downregulated genes, respectively.

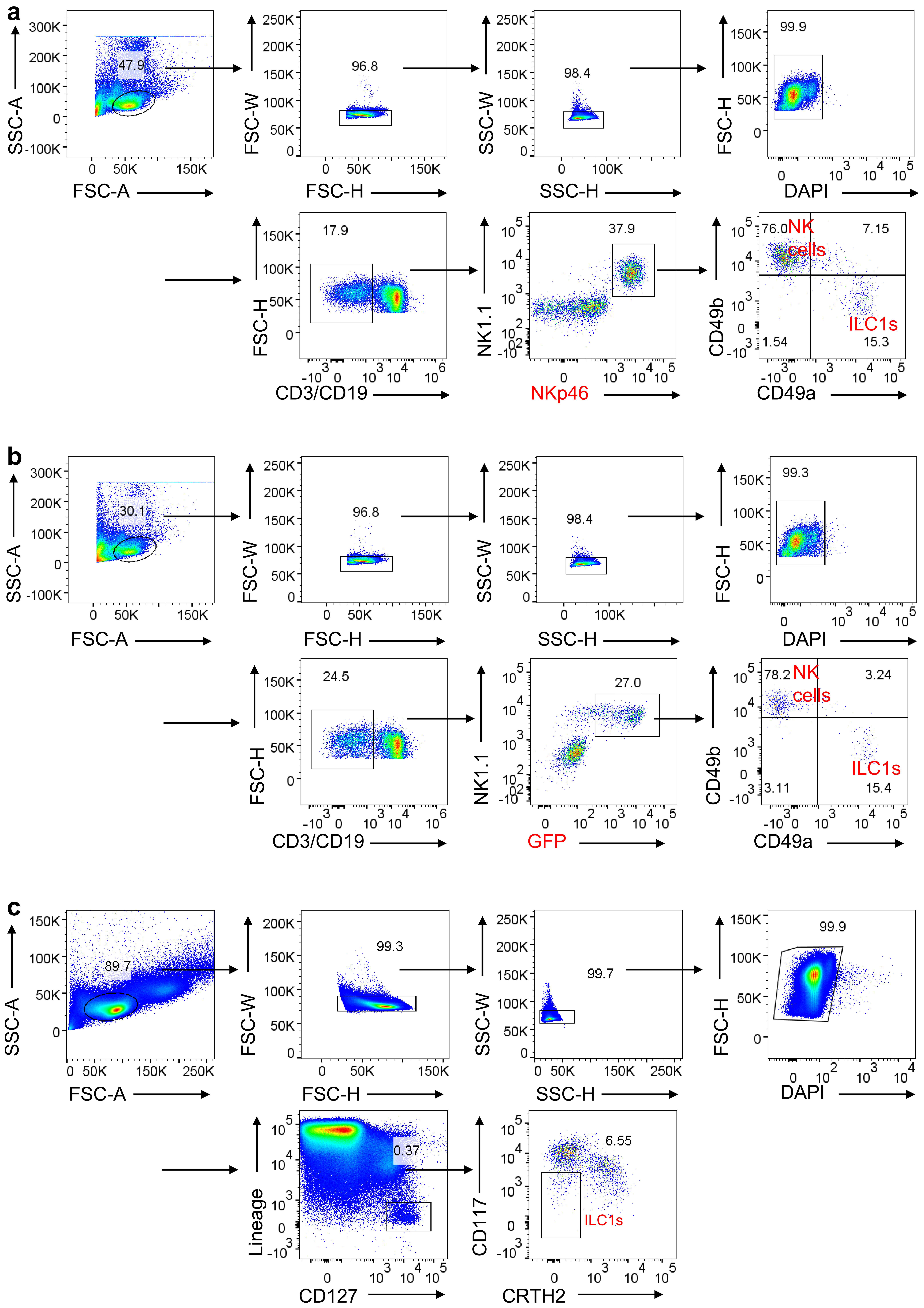

a
SSC-A
FSC-A
47.9
FSC-W
FSC-H
96.8
SSC-W
SSC-H
98.4
FSC-H
DAPI
99.9
FSC-H
CD3/CD19
17.9
NK1.1
NKp46
37.9
CD49b
CD49a
76.0
NK cells
7.15
ILC1s
1.54
15.3
b
SSC-A
FSC-A
30.1
FSC-W
FSC-H
96.8
SSC-W
SSC-H
98.4
FSC-H
DAPI
99.3
FSC-H
CD3/CD19
24.5
NK1.1
GFP
27.0
CD49b
CD49a
78.2
NK cells
3.24
ILC1s
3.11
15.4
c
SSC-A
FSC-A
89.7
FSC-W
FSC-H
99.3
SSC-W
SSC-H
99.7
FSC-H
DAPI
99.9
Lineage
CD127
0.37
CD117
CRTH2
6.55
ILC1s

Supplementary Fig. 2 The gating strategy of ILC1s. (a) The gating strategy of ILC1s isolated from the liver of *Ncr1*$^{+/+}$ mice. (b) The gating strategy of ILC1s isolated from the liver of *Ncr1*$^{gfp/gfp}$ mice. (c) The gating strategy of human ILC1s isolated from the peripheral blood mononuclear cells (PBMCs) of healthy donors. The gating panels in (a) and (b) correspond to Fig. 1a–1g, Fig. 2c–2g, Fig. 3a, 3b, 3d, 3e, Fig. 4a–4c, Fig. 5b–5i, Fig. 6b–6g. The gating panel in (c) corresponds to Fig. 7a–7e and Fig. 7i.

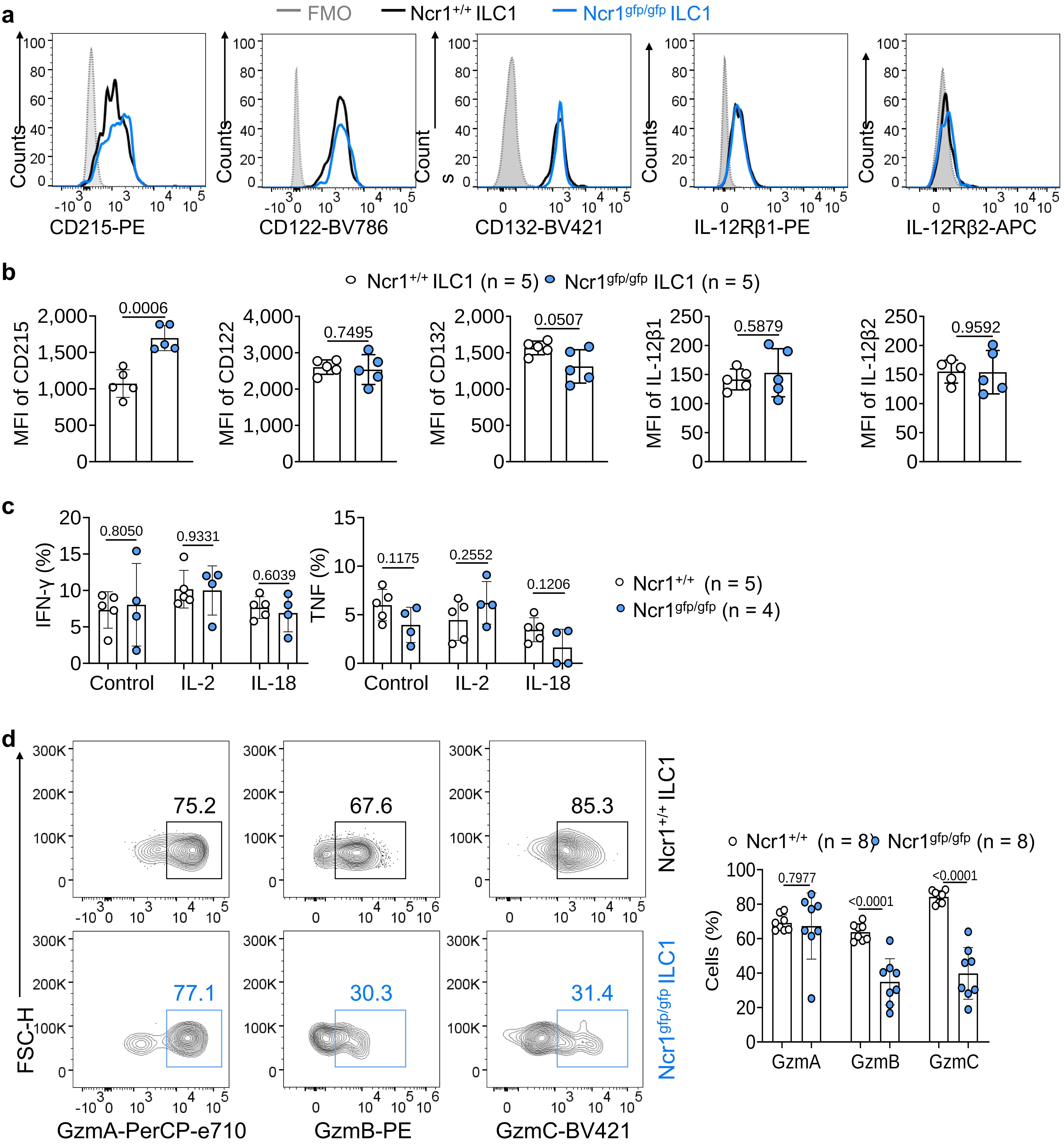

a
FMO
Ncr1+/+ ILC1
Ncr1gfp/gfp ILC1
Counts
CD215-PE
CD122-BV786
CD132-BV421
IL-12Rβ1-PE
IL-12Rβ2-APC
b
Ncr1+/+ ILC1 (n = 5)
Ncr1gfp/gfp ILC1 (n = 5)
MFI of CD215
0.0006
MFI of CD122
0.7495
MFI of CD132
0.0507
MFI of IL-12β1
0.5879
MFI of IL-12β2
0.9592
c
IFN-γ (%)
0.8050
0.9331
0.6039
TNF (%)
0.1175
0.2552
0.1206
Control
IL-2
IL-18
Ncr1+/+ (n = 5)
Ncr1gfp/gfp (n = 4)
d
FSC-H
75.2
67.6
85.3
77.1
30.3
31.4
Ncr1+/+ ILC1
Ncr1gfp/gfp ILC1
GzmA-PerCP-e710
GzmB-PE
GzmC-BV421
Ncr1+/+ (n = 8)
Ncr1gfp/gfp (n = 8)
Cells (%)
0.7977
<0.0001
<0.0001
GzmA
GzmB
GzmC

Supplementary Fig. 3 The expression and function of IL-15 and IL-12 receptors as well as the expression of granzymes in ILC1s. (a) Representative histograms showing expression of IL-15Rα (CD215), CD122, CD132, IL-12Rβ1, and IL-12Rβ2 on mouse liver ILC1s. (b) Summary data of (a) (n = 5). (c) $Ncr1^{+/+}$ ILC1s and $Ncr1^{gfp/gfp}$ ILC1s isolated from the liver of $Ncr1^{+/+}$ and $Ncr1^{gfp/gfp}$ mice, respectively, were cultured with or without IL-2 (1000 IU/ml) or IL-18 (10 ng/ml) for 4 h. Statistics of the IFN-γ^{+} (left) and TNF^{+} (right) of ILC1s ($Ncr1^{+/+}$ mice: n = 5; $Ncr1^{gfp/gfp}$ mice n = 4). (d) $Ncr1^{+/+}$ ILC1s and $Ncr1^{gfp/gfp}$ ILC1s were isolated from the liver of $Ncr1^{+/+}$ and $Ncr1^{gfp/gfp}$ mice. The production of granzymes by untreated ILC1s was checked by flow cytometry. Representative flow dot plots (left) and statistics (right) of the $GzmA^{+}$, $GzmB^{+}$, and $GzmC^{+}$ ILC1s ($Ncr1^{+/+}$ mice: n = 8; $Ncr1^{gfp/gfp}$ mice n = 8). Data are presented as mean ± s.d.; *P* values were calculated by two-tailed Student's *t* test (b, c, and d). NS, not significant. Source data are provided as a Source Data file.

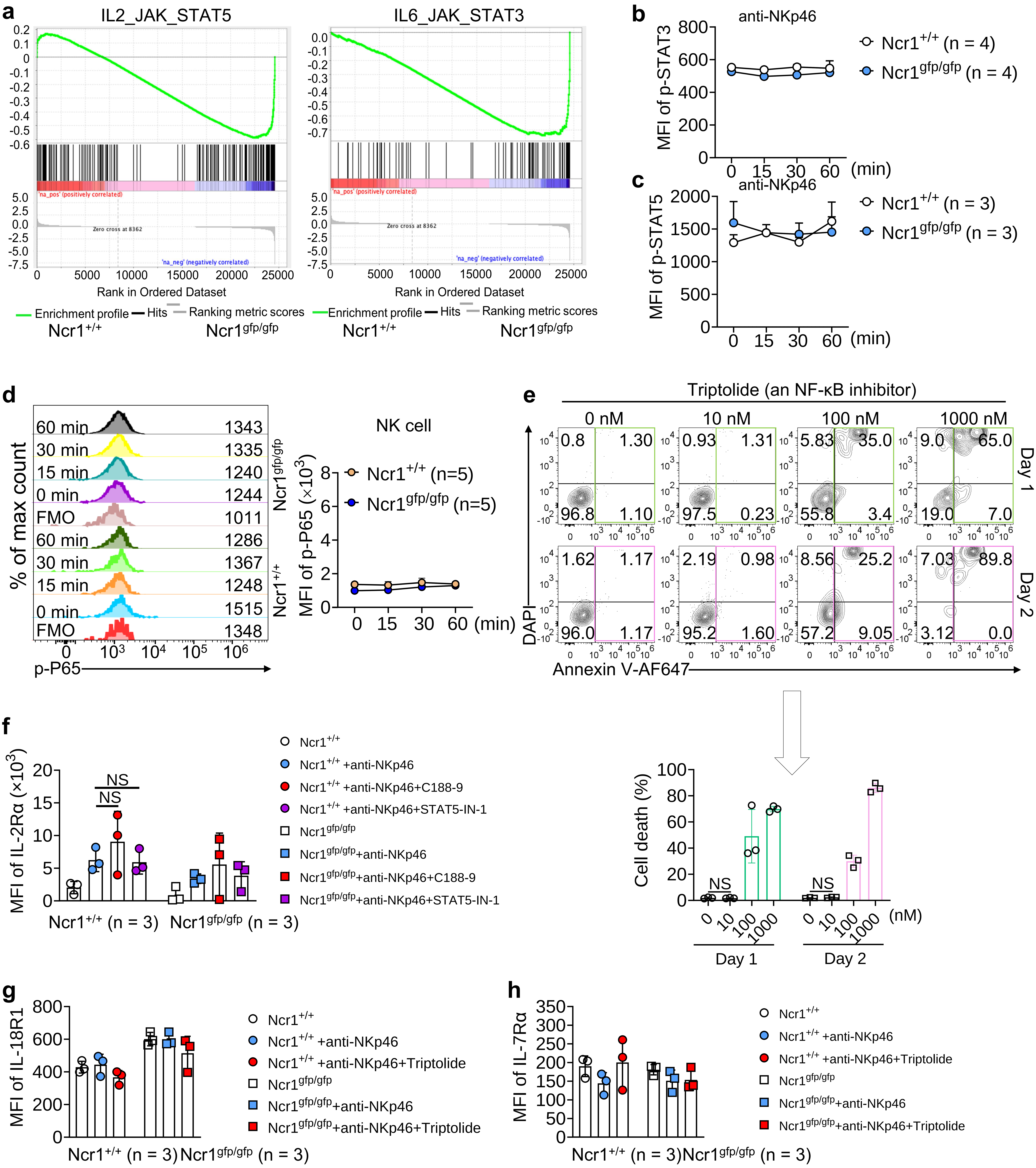
a
IL2_JAK_STAT5
IL6_JAK_STAT3
Enrichment profile
Hits
Ranking metric scores
Rank in Ordered Dataset
'na_pos' (positively correlated)
Zero cross at 8362
'na_neg' (negatively correlated)
Ncr1+/+
Ncr1gfp/gfp
b
anti-NKp46
MFI of p-STAT3
Ncr1+/+ (n = 4)
Ncr1gfp/gfp (n = 4)
(min)
c
anti-NKp46
MFI of p-STAT5
Ncr1+/+ (n = 3)
Ncr1gfp/gfp (n = 3)
(min)
d
% of max count
60 min 1343
30 min 1335
15 min 1240
0 min 1244
FMO 1011
60 min 1286
30 min 1367
15 min 1248
0 min 1515
FMO 1348
p-P65
NK cell
MFI of p-P65 (×10³)
Ncr1+/+ (n=5)
Ncr1gfp/gfp (n=5)
(min)
e
Triptolide (an NF-κB inhibitor)
0 nM
10 nM
100 nM
1000 nM
Day 1
Day 2
DAPI
Annexin V-AF647
f
MFI of IL-2Rα (×10³)
NS
Ncr1+/+ (n = 3)
Ncr1gfp/gfp (n = 3)
Ncr1+/+
Ncr1+/+ +anti-NKp46
Ncr1+/+ +anti-NKp46+C188-9
Ncr1+/+ +anti-NKp46+STAT5-IN-1
Ncr1gfp/gfp
Ncr1gfp/gfp+anti-NKp46
Ncr1gfp/gfp+anti-NKp46+C188-9
Ncr1gfp/gfp+anti-NKp46+STAT5-IN-1
Cell death (%)
NS
(nM)
Day 1
Day 2
g
MFI of IL-18R1
Ncr1+/+ (n = 3)
Ncr1gfp/gfp (n = 3)
Ncr1+/+
Ncr1+/+ +anti-NKp46
Ncr1+/+ +anti-NKp46+Triptolide
Ncr1gfp/gfp
Ncr1gfp/gfp+anti-NKp46
Ncr1gfp/gfp+anti-NKp46+Triptolide
h
MFI of IL-7Rα
Ncr1+/+ (n = 3)
Ncr1gfp/gfp (n = 3)
Ncr1+/+
Ncr1+/+ +anti-NKp46
Ncr1+/+ +anti-NKp46+Triptolide
Ncr1gfp/gfp
Ncr1gfp/gfp+anti-NKp46
Ncr1gfp/gfp+anti-NKp46+Triptolide

Supplementary Fig. 4 Engagement of anti-NKp46 antibody fails to induce activation of the JAK/STAT3/5 signaling pathway and expression of IL-18R1 and IL-7Rα in mouse ILC1s. (a) *Ncr1*$^{+/+}$ ILC1s and *Ncr1*$^{gfp/gfp}$ ILC1s were sorted for RNA-seq from the liver of *Ncr1*$^{+/+}$ mice and *Ncr1*$^{gfp/gfp}$ mice, respectively, using FACS. Gene set enrichment analysis (GSEA) plots show enrichment of the indicated target genes in the JAK-STAT5 (left) and JAK-STAT3 (right) signaling pathways in ILC1s. The X-axis shows the rank orders (*Ncr1*$^{gfp/gfp}$ ILC1s vs. *Ncr1*$^{+/+}$ ILC1s) of all the target genes (n = 5). (b and c) *Ncr1*$^{+/+}$ ILC1s and *Ncr1*$^{gfp/gfp}$ ILC1s were treated with or without anti-NKp46 antibody (5 μg/ml) for indicated times. The phosphorylation of STAT3 and STAT5 was measured by flow cytometry. Quantification of p-STAT3 (b) and p-STAT5 (c) mean fluorescence intensity (MFI) in *Ncr1*$^{+/+}$ ILC1s and *Ncr1*$^{gfp/gfp}$ ILC1s at the indicated time points (b: n = 4; c: n = 3). (d) Representative histograms (left) and statistics (right) of the level of p-P65 on mouse liver NK cells (n = 5). (e) ILC1s isolated from the liver of mice were treated with various doses of Triptolide for indicated days. Representative histograms (top) and quantification (bottom) of the percentage of ILC1 cell death (n = 3). (f) *Ncr1*$^{+/+}$ ILC1s and *Ncr1*$^{gfp/gfp}$ ILC1s were treated with anti-NKp46 antibody (5 μg/ml) in the presence or absence of STAT3 inhibitor (10 nM, C188-9) or STAT5 inhibitor (10 nM, STAT5-IN-1) for 3 days. Quantification of IL-2Rα MFI on *Ncr1*$^{+/+}$ ILC1s and *Ncr1*$^{gfp/gfp}$ ILC1s (n = 3). (g and h) *Ncr1*$^{+/+}$ ILC1s and *Ncr1*$^{gfp/gfp}$ ILC1s were treated with anti-NKp46 antibody (5 μg/ml) in the presence or absence of Triptolide (10 nM) for 3 days. Quantification of IL-18R1 (g) and IL-7Rα (h) MFI on *Ncr1*$^{+/+}$ ILC1s and *Ncr1*$^{gfp/gfp}$ ILC1s are shown (n = 3). Data are presented as mean ± s.d.; *P* values were calculated by one-way ANOVA models with adjustments (e and f). NS, not significant. Source data are provided as a Source Data file.

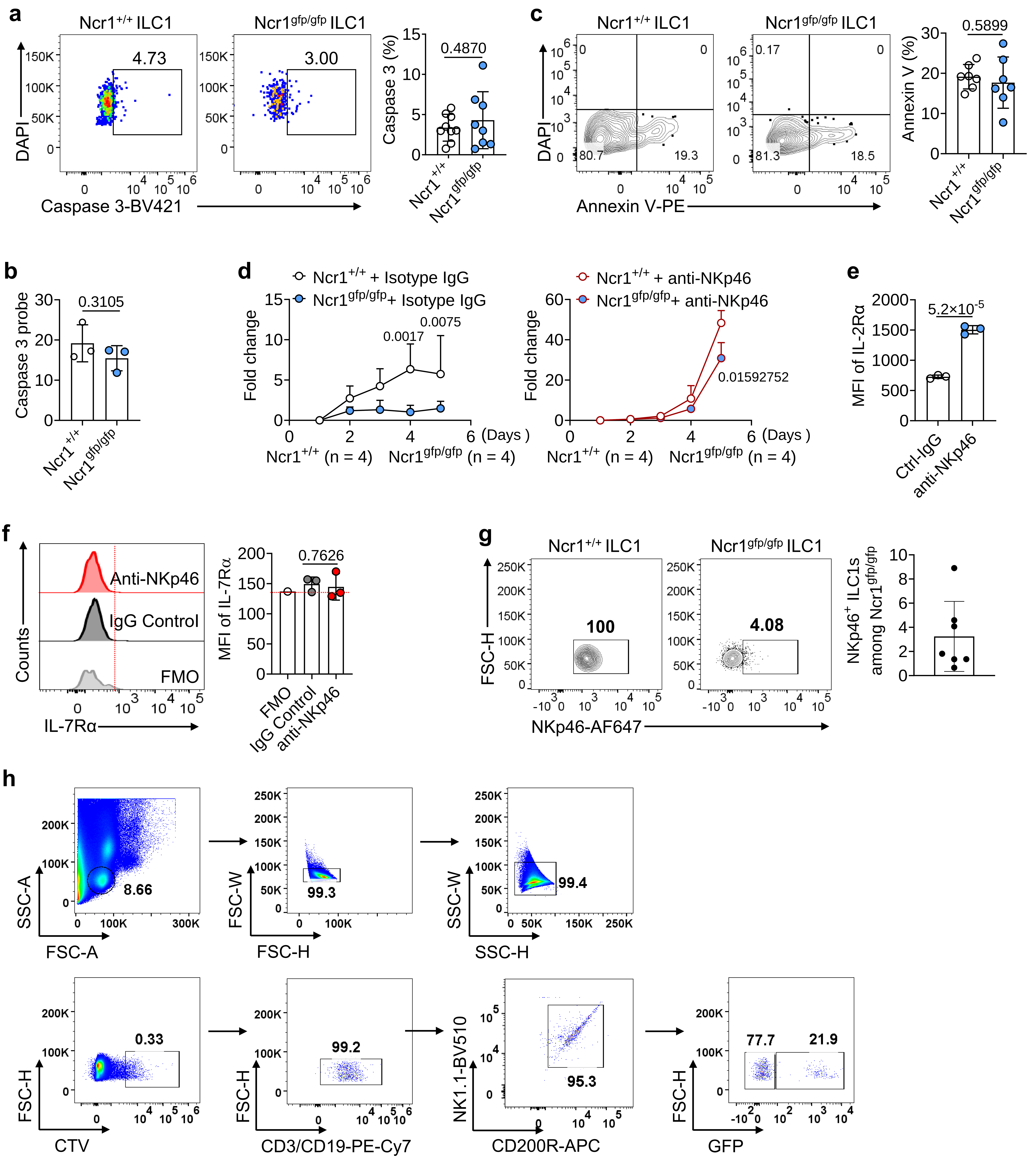

a
Ncr1+/+ ILC1
Ncr1gfp/gfp ILC1
4.73
3.00
DAPI
Caspase 3-BV421
Caspase 3 (%)
0.4870
Ncr1+/+
Ncr1gfp/gfp
c
Ncr1+/+ ILC1
Ncr1gfp/gfp ILC1
0
0
80.7
19.3
0.17
0
81.3
18.5
DAPI
Annexin V-PE
Annexin V (%)
0.5899
b
Caspase 3 probe
0.3105
d
Ncr1+/+ + Isotype IgG
Ncr1gfp/gfp+ Isotype IgG
0.0017
0.0075
Fold change
(Days)
Ncr1+/+ (n = 4)
Ncr1gfp/gfp (n = 4)
Ncr1+/+ + anti-NKp46
Ncr1gfp/gfp+ anti-NKp46
0.01592752
e
MFI of IL-2Rα
5.2×10-5
Ctrl-IgG
anti-NKp46
f
Anti-NKp46
IgG Control
FMO
Counts
IL-7Rα
MFI of IL-7Rα
0.7626
FMO
IgG Control
anti-NKp46
g
Ncr1+/+ ILC1
Ncr1gfp/gfp ILC1
100
4.08
FSC-H
NKp46-AF647
NKp46+ ILC1s among Ncr1gfp/gfp
h
8.66
SSC-A
FSC-A
99.3
FSC-W
FSC-H
99.4
SSC-W
SSC-H
0.33
FSC-H
CTV
99.2
FSC-H
CD3/CD19-PE-Cy7
95.3
NK1.1-BV510
CD200R-APC
77.7
21.9
FSC-H
GFP

Supplementary Fig. 5 NKp46 is not essential for ILC1 survival. (a) The percentages of Caspase 3^+ ILC1s in the liver of *Ncr1*$^{+/+}$ and *Ncr1*$^{gfp/gfp}$ mice were measured by flow cytometry (*Ncr1*$^{+/+}$ mice: n = 9; *Ncr1*$^{gfp/gfp}$ mice: n = 8). (b) The caspase activity of ILC1s in the liver of *Ncr1*$^{+/+}$ and *Ncr1*$^{gfp/gfp}$ mice was measured by flow cytometry (n = 3). (c) Statistics of annexin$^+$ ILC1s isolated from the livers of mice (n = 7). (d) *Ncr1*$^{+/+}$ ILC1s and *Ncr1*$^{gfp/gfp}$ ILC1s sorted from the liver of *Ncr1*$^{+/+}$ and *Ncr1*$^{gfp/gfp}$ mice, respectively, were cultured without (left) or with anti-NKp46 antibody (right; 5 μg/ml) in the presence of IL-2 (1000 IU/ml) and IL-7 (100 ng/ml) for 5 days. Statistics of the fold change in the number of ILC1s (*Ncr1*$^{+/+}$ mice: n = 4; *Ncr1*$^{gfp/gfp}$ mice: n = 4). (e) Statistics of MFI of IL-2Rα on the liver ILC1s of *Ncr1*$^{+/+}$ mice treated with or without anti-NKp46 (n = 3). (f) Representative histogram (left) and statistics of (right) IL-7Rα (n = 3). (g) Representative of the expression of NKp46 on the *Ncr1*$^{gfp/gfp}$ positive cells isolated from the *Ncr1*$^{gfp/gfp}$ mice (n = 7). (h) *Ncr1*$^{+/+}$ (GFP negative) or *Ncr1*$^{gfp/gfp}$ (GFP positive) ILC1s were sorted from the liver of mice, mixed at a ratio of 1:1, and then *i.v.* injected into *Rag2*$^{-/-}$*γc*$^{-/-}$ mice. The gating strategy of ILC1s in the liver of *Rag2*$^{-/-}$*γc*$^{-/-}$ mice is shown. Data are presented as mean ± s.d.; *P* values were calculated by two-tailed Student's *t* test (a, b, c, e, and f) or linear mixed models with adjustments (d). NS, not significant. Source data are provided as a Source Data file.

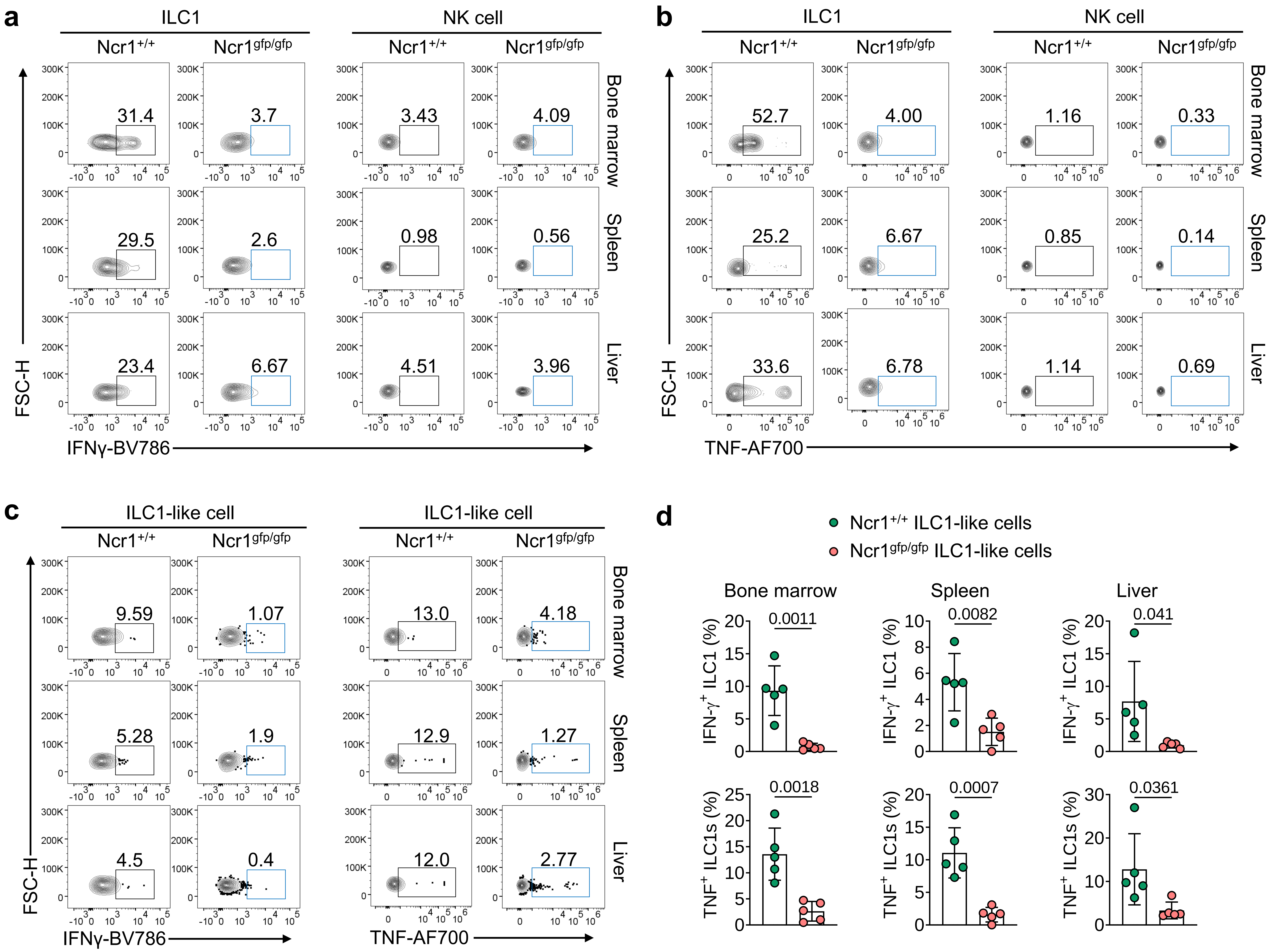
a
ILC1
NK cell
Ncr1+/+
Ncr1gfp/gfp
Bone marrow
Spleen
Liver
31.4
3.7
3.43
4.09
29.5
2.6
0.98
0.56
23.4
6.67
4.51
3.96
FSC-H
IFNγ-BV786
b
52.7
4.00
1.16
0.33
25.2
6.67
0.85
0.14
33.6
6.78
1.14
0.69
TNF-AF700
c
ILC1-like cell
9.59
1.07
13.0
4.18
5.28
1.9
12.9
1.27
4.5
0.4
12.0
2.77
d
Ncr1+/+ ILC1-like cells
Ncr1gfp/gfp ILC1-like cells
IFN-γ+ ILC1 (%)
TNF+ ILC1s (%)
0.0011
0.0082
0.041
0.0018
0.0007
0.0361

Supplementary Fig. 6 Production of IFN-γ and TNF by ILC1s and ILC1-like cells from *Ncr1^gfp/gfp^* mice is impaired compared to those from *Ncr1^+/+^* mice after both types of mice are implanted with C1498. Representative flow cytometry plots showing the percentage of IFN-γ^+ ILC1s and NK cells (a), TNF^+ ILC1s and NK cells (b) as well as IFN-γ^+ and TNF^+ ILC1-like cells (c and d). All cells were isolated from the bone marrow, the spleen, or the liver of mice implanted with C1498. Data are presented as mean ± s.d.; *P* values were calculated by two-tailed Student's *t* test (d). Source data are provided as a Source Data file.

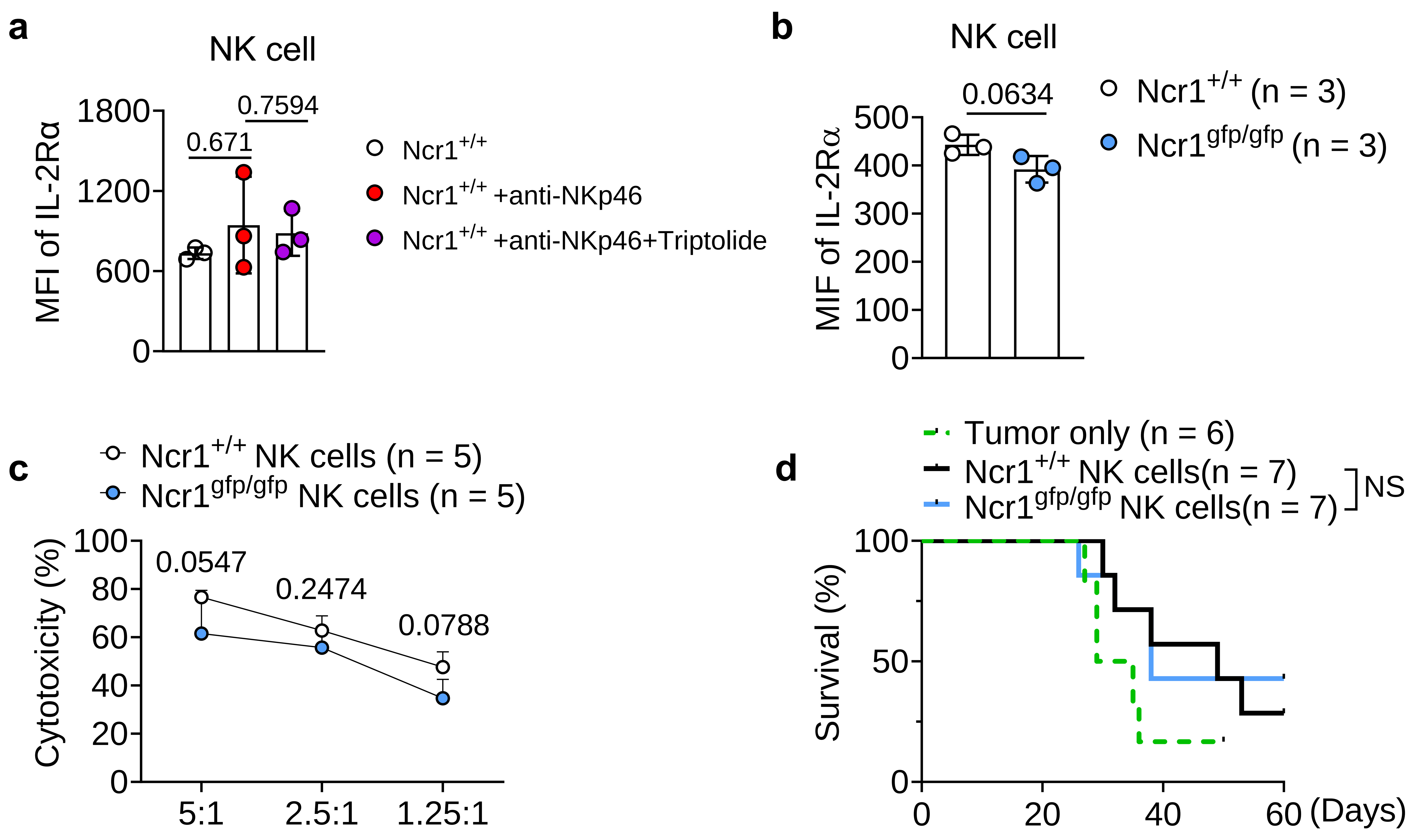
a
NK cell
MFI of IL-2Rα
1800
1200
600
0
0.671
0.7594
Ncr1+/+
Ncr1+/+ +anti-NKp46
Ncr1+/+ +anti-NKp46+Triptolide
b
NK cell
MIF of IL-2Rα
500
400
300
200
100
0
0.0634
Ncr1+/+ (n = 3)
Ncr1gfp/gfp (n = 3)
c
Ncr1+/+ NK cells (n = 5)
Ncr1gfp/gfp NK cells (n = 5)
Cytotoxicity (%)
100
80
60
40
20
0
0.0547
0.2474
0.0788
5:1
2.5:1
1.25:1
d
Tumor only (n = 6)
Ncr1+/+ NK cells(n = 7)
Ncr1gfp/gfp NK cells(n = 7)
NS
Survival (%)
100
50
0
0
20
40
60
(Days)

Supplementary Fig. 7 Loss of NKp46 does not affect the expression of IL-2Rα and the cytotoxicity of NK cells in vitro and in vivo. (a) *Ncr1*$^{+/+}$ NK cells were treated with or without anti-NKp46 antibody (5 μg/ml) for 3 days in the presence or absence of Triptolide. Quantification of IL-2Rα expression on NK cells is shown (n = 3). (b) Expression of IL-2Rα on *Ncr1*$^{+/+}$ and *Ncr1*$^{gfp/gfp}$ NK cells was measured by flow cytometry (n = 3). (c) NK cells sorted from the liver of *Ncr1*$^{+/+}$ and *Ncr1*$^{gfp/gfp}$ mice were cultured with C1498 at the indicated ratios for 24 h. Luciferase activity in the wells with tumor cells was measured with a luminescence microplate reader (n = 5). (d) *Ncr1*$^{+/+}$ and *Ncr1*$^{gfp/gfp}$ mice were i.v. co-injected with 1.0×10^6 C1498-Luc AML cells, followed by the treatment with 4.0×10^6 *Ncr1*$^{+/+}$ or *Ncr1*$^{gfp/gfp}$ NK cells. Survival of the mice injected with *Ncr1*$^{+/+}$ and *Ncr1*$^{gfp/gfp}$ NK cells was monitored. Survival data were analyzed by Kaplan-Meier survival analysis and log-rank test (Tumor only: n = 6; *Ncr1*$^{+/+}$ and *Ncr1*$^{gfp/gfp}$ mice: n = 7). Data are presented as mean ± s.d.; *P* values were calculated by two-tailed Student's *t* test (a and b) or linear mixed models with adjustments(c). NS, not significant. Source data are provided as a Source Data file.
