# Supplementary material for: NKp46 enhances type 1 innate lymphoid cell proliferation and function and anti-acute myeloid leukemia activity
Source: Nat Commun. 2025 Jan 24;16:989. doi: 10.1038/s41467-025-55923-w (PMC11760942; doi:10.1038/s41467-025-55923-w)
Supplement: Supplementary file 2 — Reporting Summary [file 41467_2025_55923_MOESM2_ESM.pdf]

nature portfolio

Corresponding author(s): Michael A. Caligiuri

Last updated by author(s): Dec 13, 2024

Reporting Summary

Nature Portfolio wishes to improve the reproducibility of the work that we publish. This form provides structure for consistency and transparency in reporting. For further information on Nature Portfolio policies, see our Editorial Policies and the Editorial Policy Checklist.

Statistics

For all statistical analyses, confirm that the following items are present in the figure legend, table legend, main text, or Methods section.

n/a	Confirmed	
☐	☒	The exact sample size (*n*) for each experimental group/condition, given as a discrete number and unit of measurement
☐	☒	A statement on whether measurements were taken from distinct samples or whether the same sample was measured repeatedly
☐	☒	The statistical test(s) used AND whether they are one- or two-sided<br>*Only common tests should be described solely by name; describe more complex techniques in the Methods section.*
☒	☐	A description of all covariates tested
☐	☒	A description of any assumptions or corrections, such as tests of normality and adjustment for multiple comparisons
☐	☒	A full description of the statistical parameters including central tendency (e.g. means) or other basic estimates (e.g. regression coefficient) AND variation (e.g. standard deviation) or associated estimates of uncertainty (e.g. confidence intervals)
☐	☒	For null hypothesis testing, the test statistic (e.g. *F*, *t*, *r*) with confidence intervals, effect sizes, degrees of freedom and *P* value noted<br>*Give P values as exact values whenever suitable.*
☒	☐	For Bayesian analysis, information on the choice of priors and Markov chain Monte Carlo settings
☒	☐	For hierarchical and complex designs, identification of the appropriate level for tests and full reporting of outcomes
☒	☐	Estimates of effect sizes (e.g. Cohen's *d*, Pearson's *r*), indicating how they were calculated

Our web collection on statistics for biologists contains articles on many of the points above.

Software and code

Policy information about availability of computer code

Data collection	BD LSRFortessaTM X20 and BD FACSAriaTM Fusion were used for flow cytometry data collection. Zeiss AxioCam 702 sCMOS Mono was used for acquisition of microscopy data.
Data analysis	Flow Cytometry data were analyzed by FlowJo V10 (Treestar) and Novoexpress (Agilent); Statistical analysis all conducted using GraphPad Prism V9 and SAS v.9.4. Trimmomatic (v.0.38), FASTP(v.0.19.4), RseQC(v.2.5), edgeR (v.3.28.1), GSEA (v.3.0), and Cluster (v.3.0) were used for RNA-sequencing analysis.

For manuscripts utilizing custom algorithms or software that are central to the research but not yet described in published literature, software must be made available to editors and reviewers. We strongly encourage code deposition in a community repository (e.g. GitHub). See the Nature Portfolio guidelines for submitting code & software for further information.

Data

Policy information about availability of data

All manuscripts must include a data availability statement. This statement should provide the following information, where applicable:

- Accession codes, unique identifiers, or web links for publicly available datasets
- A description of any restrictions on data availability
- For clinical datasets or third party data, please ensure that the statement adheres to our policy

Source data are provided with this paper. The RNA-seq data used in this study are accessible in GEO under accession code GSE283199. All other data generated during this study are available upon request.

Research involving human participants, their data, or biological material

Policy information about studies with human participants or human data. See also policy information about sex, gender (identity/presentation), and sexual orientation and race, ethnicity and racism.

Reporting on sex and gender	Recruitment for this cohort was independent of sex, allowing any healthy donor or patient with newly diagnosed, relapsed, or refractory AML to be eligible. Due to the limited size of the cohort, we did not perform any analyses stratified by sex.
Reporting on race, ethnicity, or other socially relevant groupings	Information on race, ethnicity or other socio-economical parameters were not collected for this cohort.
Population characteristics	All participants in this study were either healthy donors or patients with relapsed or refractory acute myeloid leukemia. Eligibility criteria required individuals to be at least 18 years old and to provide written informed consent in accordance with national legal and regulatory requirements prior to any project-specific procedures.
Recruitment	Patients who met the inclusion criteria outlined above were recruited for this study, so we do not anticipate any self-selection or other biases.
Ethics oversight	This study was approved by the ethics committee of the City of Hope National Medical Center.

Note that full information on the approval of the study protocol must also be provided in the manuscript.

Field-specific reporting

Please select the one below that is the best fit for your research. If you are not sure, read the appropriate sections before making your selection.

☒ Life sciences ☐ Behavioural & social sciences ☐ Ecological, evolutionary & environmental sciences

For a reference copy of the document with all sections, see nature.com/documents/nr-reporting-summary-flat.pdf

Life sciences study design

All studies must disclose on these points even when the disclosure is negative.

Sample size	A reasonable sample sizes were determined based on our and other investigators experience and similar research reported in the literature to ensure adequate reproducibility of results. The sample size and associated statistics are indicated in the figures and respective legends.
Data exclusions	No data were excluded from the analyses.
Replication	All experiments were reliably reproduced and results are represented as mean +/-SD as appropriate, which is indicated in figure legends. One-way ANOVA model was utilized to compare three or more conditions. Experiments were repeated with at least two to three biologically independent for all results presented in the manuscript. When needed, P values were adjusted for multiple comparisons using Holm's or the Bonferroni method procedure. A P value of 0.05 or less was considered statistically significant, which is described in the methods section of the main text.
Randomization	Peripheral blood cones sed to isolate healthy ILC1s and peripheral blood from patients with AML used to isolate AML ILC1s were de-identified and randomly picked up. No sex and/or gender was considered in the study design and sex and/or gender of participants was determined based on self-report or assigned (and methodology used). In animal study, six- to eight-week-old mice were matched by age and sex and randomly assigned to specific treatment groups.
Blinding	Experimenters were blinded to observe survival of mice. Otherwise, blinding was not performed, such as during in vitro experiments, where experimenters were required to know the conditions of each well.

Reporting for specific materials, systems and methods

We require information from authors about some types of materials, experimental systems and methods used in many studies. Here, indicate whether each material, system or method listed is relevant to your study. If you are not sure if a list item applies to your research, read the appropriate section before selecting a response.

Materials & experimental systems

n/a	Involved in the study
	☒ Antibodies
	☒ Eukaryotic cell lines
☒	Palaeontology and archaeology
	☒ Animals and other organisms
☒	Clinical data
☒	Dual use research of concern
☒	Plants

Methods

n/a	Involved in the study
☒	ChIP-seq
	☒ Flow cytometry
☒	MRI-based neuroimaging

Antibodies

Antibodies used

Anti-Mouse CD3e-PE-Cy7 BD Biosciences Cat# 552774, Clone: 145-2C11; RRID:AB_394460
Anti-Mouse CD19-PE-Cy7 BD Biosciences Cat# 552854, Clone: 1D3; RRID:AB_394495
Anti-Mouse CD335 (NKp46)-AF647 BD Biosciences Cat# 560755, Clone: 29A1.4; RRID:AB_1727464
Anti-Mouse NK1.1-BV510 BD Biosciences Cat# 563096, Clone: PK136; RRID:AB_2738002
Anti-Mouse NK1.1-FITC BD Biosciences Cat# 561082, Clone: PK136; RRID:AB_394676
Anti-Mouse CD49b-PE BD Biosciences Cat# 553858, Clone: DX5; RRID:AB_395094
Anti-Mouse CD49b-BUV395 BD Biosciences Cat# 740250, Clone: HMα2; RRID:AB_2739996
Anti-Rat/Mouse CD49a-BV711 BD Biosciences Cat# 564863, Clone: Ha31/8; RRID: AB_2738987
Anti-Mouse IFN-γ-BV786 BD Biosciences Cat# 563773, Clone: XMG1.2; RRID:AB_2738419
Anti-Mouse TNF-α-AF700 BioLegend Cat# 506338, Clone: MP6-XT22; RRID:AB_2562918
Anti-Mouse CD127 (IL-7Rα)-BV421 BioLegend Cat# 121127, Clone: SB/199; RRID: AB_3106223
Anti-Mouse CD127 (IL-7Rα)-PerCP/Cyanine5.5 BioLegend Cat# 121114, Clone: SB/199; RRID:AB_1134206
Anti-Mouse IL-18R1-PerCP-e710 ThermoFisher Cat #46-5183-82, Clone: P3TUNYA; RRID: AB_2573764
Anti-Mouse CD25-BV605 Biolegend Cat# 102036, Clone: PC61; RRID: AB_11126977
Anti-Mouse CD215-PE Biolegend Cat# 153504, Clone: 6B4C88; RRID: AB_2721341
Anti-Mouse CD122-BV786 BD Biosciences Cat# 740908, Clone: 5H4; RRID: AB_2740552
Anti-Mouse CD132-BV421 BD Biosciences Cat# 740037, Clone: 4G3; RRID: AB_2739807
Anti-Mouse IL-12Rβ1-PE BD Biosciences Cat# 551974, Clone: 114; RRID: AB_394310
Anti-Mouse IL-12Rβ2-APC R&D Cat# FAB1959A-025, Clone: 305719; RRID: AB_2124049
Anti-Mouse CD200R1-APC Thermofisher Cat# 17-5201-82, Clone# OX110; RRID: AB_10717289
Anti-Granzyme A-PerCP-eFluor™ 710 Thermofisher Cat# 46-5831-82, Clone: GzA-3G8.5; RRID: AB_2573775
Anti-Granzyme B-PE Biolegend Cat# 372208, Clone: QA16A02; RRID: AB_2687031
Anti-Granzyme C-BV421 BD Biosciences Cat# 569861, Clone: SFC1D8.rMAb; RRID: NO
Brilliant Violet 421™ anti-STAT3 Phospho (Tyr705) Antibody Biolegend, Cat# 651010, Clone: 13A3-1; RRID: AB_2572087
PE anti-STAT3 Phospho (Ser727) Antibody Biolegend Cat# 698906, Clone: A16089B; RRID: AB_2721646
Phospho-Stat5 (Tyr694) (D47E7) XP® Rabbit mAb (PE Conjugate), Cell Signaling Technology Cat# 14603, Clone: D47E7; RRID: AB_AB_2798533
PE anti-p38 MAPK Phospho (Thr180/Tyr182) Recombinant Antibody Biolegend, Cat# 690254, Clone: A16016A.Rec;RRID:AB_3097635
Anti-Human CD3-FITC BD Biosciences Cat# 561802, Clone: HIT3a; RRID:AB_395745
Anti-Human CD4-FITC BD Biosciences Cat# 555346, Clone: RPA-T4; RRID:AB_395751
Anti-Human CD8-FITC BD Biosciences Cat# 555634, Clone: HIT8a; RRID:AB_395996
Anti-Human CD14-FITC BD Biosciences Cat# 555397, Clone: M5E2; RRID:AB_395798
Anti-Human CD15-FITC BD Biosciences Cat# 555401, Clone: HI98; RRID:AB_395801
Anti-Human CD16-FITC BD Biosciences Cat# 555406, Clone: 3G8; RRID:AB_395806
Anti-Human CD19-FITC BD Biosciences Cat# 555412, Clone: HIB19; RRID:AB_395812
Anti-Human CD20-FITC BD Biosciences Cat# 555622, Clone: 2H7; RRID:AB_395988
Anti-Human CD33-FITC BD Biosciences Cat# 555626, Clone: HIM3-4; RRID:AB_395992
Anti-Human CD34-FITC BD Biosciences Cat# 555821, Clone: 581; RRID:AB_396150
Anti-Human CD203C (NP4D6)-FITC Thermo Fisher Scientific Cat# MA5-28586, Clone: NP4D6; RRID:AB_2745545
Anti-Human FcεRIα-FITC Biolegend Cat# 334608, Clone: AER-37 (CRA-1); RRID:AB_1227654
Anti-Human CD56 (NCAM-1)-AF700 BD Biosciences Cat# 557919, Clone: B159; RRID:AB_396940
Anti-Human CD56 (NCAM-1)-FITC BD Biosciences Cat# 562794, Clone: B159; RRID:AB_2737799
Anti-Human CD56-BV421 BD Biosciences Cat# 562751, Clone: NCAM16.2; RRID:AB_2732054
Anti-Human CD127-APC BD Biosciences Cat# 558598, Clone: HIL-7R-M21; RRID:AB_647113
Anti-Human CD117-PE BD Biosciences Cat# 555714, Clone: YB5.B8; RRID:AB_396058
Anti-Human CD294 (CRTH2)-PE-Cy7 Biolegend Cat# 350118, Clone: BM16; RRID:AB_2562470
Anti-Human NKp46-BV605 BD Bioscience Cat# 743710, clone: 9E2/NKp46; RRID:AB_2741689
7-AAD Staining Solution 2mL antibody BD Biosciences Cat# 559925, RRID:AB_2869266
DAPI Solution BD Biosciences Cat# 564907, RRID:AB_2869624
Violet Live Cell Caspase Probe BD Biosciences Cat# 565521, RRID: AB_2869682

Annexin V-PE Biolegend Cat# 640947, RRID: NO
Anti-Human TNF BV650 Biolegend Cat# 502938, Clone: MAb11; RRID: AB_2561355
Anti-Human IFN-γ -BV421 Biolegend Cat# 506538 , Clone: B27; RRID: AB_2801097
Phospho-NF-κB p65 (Ser536) (93H1) Rabbit mAb (PE Conjugate) Cell signaling Technology Cat# 5733, Clone: 93H1; RRID: AB_10706937
PE anti-NF-κB p65 Antibody Biolegend Cat# 653004, Clone: 14G10A21; RRID: AB_2562768
BD Horizon™ BUV395 Mouse Anti-Ki-67 BD Biosciences Cat# 564071, Clone: B56; RRID:AB_2738577
BD Horizon™ BV421 Rabbit Anti-Active Caspase-3 BD Biosciences Cat# 570786, Clone: C92-605.rMAb; RRID: NO
Rat IgG2a kappa Isotype Control (eBR2a) Thermofisher Cat# 16-4321-82, clone: eBR2a; RRID: AB_470156
CD335 (NKp46) Monoclonal Antibody (29A1.4) Thermofisher Cat# 16-3351-81, Clone: 29A1.4; RRID: AB_1724164
NKp46 Polyclonal Antibody Thermofisher Cat# PA5-46986, clone: Polyclonal; RRID: AB_2606904
Ultra-LEAF™ Purified anti-mouse CD314 (NKG2D) Antibody Biolegend Cat# 115713, Clone: C7; RRID: AB_2832393
BD Horizon™ BV510 Mouse anti-BrdU BD Biosciences Cat# 563445, Clone: 3D4; RRID: AB_2738210

Validation

All antibodies commercially available flow cytometry antibodies for staining mouse and human samples and validated by the manufacturer.
BD Biosciences (https://www.bdbiosciences.com/en-us/reagents/research-reagents/antibodies-and-buffers)
BioLegend (https://www.biolegend.com/nl-nl/reproducibility)
Thermofisher Scientific (https://www.thermofisher.com/us/en/home/life-science/antibodies/invitrogenantibody-validation.html)
These antibodies are further validated and routinely used in our lab.

Anti-Mouse CD3e-PE-Cy7 BD Biosciences Cat# 552774, Clone: 145-2C11; RRID:AB_394460
https://www.bdbiosciences.com/en-us/products/reagents/flow-cytometry-reagents/research-reagents/single-color-antibodies-ruo/pe-cy-7-hamster-anti-mouse-cd3e.552774

Anti-Mouse CD19-PE-Cy7 BD Biosciences Cat# 552854, Clone: 1D3; RRID:AB_394495
https://www.bdbiosciences.com/en-us/products/reagents/flow-cytometry-reagents/research-reagents/single-color-antibodies-ruo/pe-cy-7-rat-anti-mouse-cd19.552854

Anti-Mouse CD335 (NKp46)-AF647 BD Biosciences Cat# 560755, Clone: 29A1.4; RRID:AB_1727464
https://www.bdbiosciences.com/en-us/products/reagents/flow-cytometry-reagents/research-reagents/single-color-antibodies-ruo/alexa-fluor-647-rat-anti-mouse-cd335-nkp46.560755

Anti-Mouse NK1.1-BV510 BD Biosciences Cat# 563096, Clone: PK136; RRID:AB_2738002
https://www.bdbiosciences.com/en-us/products/reagents/flow-cytometry-reagents/research-reagents/single-color-antibodies-ruo/bv510-mouse-anti-mouse-nk-1-1.563096

Anti-Mouse NK1.1-FITC BD Biosciences Cat# 561082, Clone: PK136; RRID:AB_394676
https://www.bdbiosciences.com/en-us/products/reagents/flow-cytometry-reagents/research-reagents/single-color-antibodies-ruo/fitc-mouse-anti-mouse-nk-1-1.561082

Anti-Mouse CD49b-PE BD Biosciences Cat# 553858, Clone: DX5; RRID:AB_395094
https://www.bdbiosciences.com/en-us/products/reagents/flow-cytometry-reagents/research-reagents/single-color-antibodies-ruo/pe-rat-anti-mouse-cd49b.553858

Anti-Mouse CD49b-BUV395 BD Biosciences Cat# 740250, Clone: HMα2; RRID:AB_2739996
https://www.bdbiosciences.com/en-us/products/reagents/flow-cytometry-reagents/research-reagents/single-color-antibodies-ruo/buv395-hamster-anti-mouse-cd49b.740250

Anti-Rat/Mouse CD49a-BV711 BD Biosciences Cat# 564863, Clone: Ha31/8; RRID: AB_2738987
https://www.bdbiosciences.com/en-us/products/reagents/flow-cytometry-reagents/research-reagents/single-color-antibodies-ruo/bv711-hamster-anti-rat-mouse-cd49a.564863

Anti-Mouse IFN-γ-BV786 BD Biosciences Cat# 563773, Clone: XMG1.2; RRID:AB_2738419
https://www.bdbiosciences.com/en-us/products/reagents/flow-cytometry-reagents/research-reagents/single-color-antibodies-ruo/bv786-rat-anti-mouse-ifn.563773

Anti-Mouse TNF-α-AF700 BioLegend Cat# 506338, Clone: MP6-XT22; RRID:AB_2562918
https://www.biolegend.com/en-ie/products/alexa-fluor-700-anti-mouse-tnf-alpha-antibody-9146

Anti-Mouse CD127 (IL-7Rα)-BV421 BioLegend Cat# 121127, Clone: SB/199; RRID: AB_3106223
https://www.biolegend.com/en-ie/products/brilliant-violet-421-anti-mouse-cd127-il-7r-antibody-25225

Anti-Mouse CD127 (IL-7Rα)-PerCP/Cyanine5.5 BioLegend Cat# 121114, Clone: SB/199; RRID:AB_1134206
https://www.biolegend.com/en-ie/products/percp-cyanine5-5-anti-mouse-cd127-il-7ralpha-antibody-4517

Anti-Mouse IL-18R1-PerCP-e710 ThermoFisher Cat #46-5183-82, Clone: P3TUNYA; RRID: AB_2573764
https://www.thermofisher.com/antibody/product/CD218a-IL-18Ra-Antibody-clone-P3TUNYA-Monoclonal/46-5183-82

Anti-Mouse CD25-BV605 Biolegend Cat# 102036, Clone: PC61; RRID: AB_11126977
https://www.biolegend.com/en-ie/products/brilliant-violet-605-anti-mouse-cd25-antibody-7639

Anti-Mouse CD215-PE Biolegend Cat# 153504, Clone: 6B4C88; RRID: AB_2721341
https://www.biolegend.com/en-ie/products/pe-anti-mouse-cd215-il-15ralpha-antibody-15123

Anti-Mouse CD122-BV786 BD Biosciences Cat# 740908, Clone: 5H4; RRID: AB_2740552
https://www.bdbiosciences.com/en-us/products/reagents/flow-cytometry-reagents/research-reagents/single-color-antibodies-ruo/bv786-rat-anti-mouse-cd122.740908

Anti-Mouse CD132-BV421 BD Biosciences Cat# 740037, Clone: 4G3; RRID: AB_2739807
https://www.bdbiosciences.com/en-us/products/reagents/flow-cytometry-reagents/research-reagents/single-color-antibodies-ruo/bv421-rat-anti-mouse-cd132.740037

Anti-Mouse IL-12Rβ1-PE BD Biosciences Cat# 551974, Clone: 114; RRID: AB_394310
https://www.bdbiosciences.com/en-us/products/reagents/flow-cytometry-reagents/research-reagents/single-color-antibodies-ruo/pe-mouse-anti-mouse-cd212.551974

Anti-Mouse IL-12Rβ2-APC R&D Cat# FAB1959A-025, Clone: 305719; RRID: AB_2124049
https://www.rndsystems.com/products/human-mouse-il-12-rbeta2-apc-conjugated-antibody-305719_fab1959a

Anti-Mouse CD200R1-APC Thermofisher Cat# 17-5201-82, Clone# OX110; RRID: AB_10717289
https://www.thermofisher.com/antibody/product/CD200-Receptor-Antibody-clone-OX110-Monoclonal/17-5201-82

Anti-Granzyme A-PerCP-eFluor™ 710 Thermofisher Cat# 46-5831-82, Clone: GzA-3G8.5; RRID: AB_2573775
https://www.thermofisher.com/antibody/product/Granzyme-A-Antibody-clone-GzA-3G8-5-Monoclonal/46-5831-82

Anti-Granzyme B-PE Biolegend Cat# 372208, Clone: QA16A02; RRID: AB_2687031
https://www.biolegend.com/en-ie/products/pe-anti-human-mouse-granzyme-b-recombinant-antibody-14431

Anti-Granzyme C-BV421 BD Biosciences Cat# 569861, Clone: SFC1D8.rMAb; RRID: NO
https://www.bdbiosciences.com/en-us/products/reagents/flow-cytometry-reagents/research-reagents/single-color-antibodies-ruo/bv421-rat-anti-mouse-granzyme-c.569861

Brilliant Violet 421™ anti-STAT3 Phospho (Tyr705) Antibody Biolegend, Cat# 651010, Clone: 13A3-1; RRID: AB_2572087
https://www.biolegend.com/en-ie/products/brilliant-violet-421-anti-stat3-phospho-tyr705-antibody-13030

PE anti-STAT3 Phospho (Ser727) Antibody Biolegend Cat# 698906, Clone: A16089B; RRID: AB_2721646
https://www.biolegend.com/en-ie/products/pe-anti-stat3-phospho-ser727-antibody-15104

Phospho-Stat5 (Tyr694) (D47E7) XP® Rabbit mAb (PE Conjugate), Cell Signaling Technology Cat# 14603, Clone: D47E7; RRID: AB_AB_2798533
https://www.cellsignal.com/products/antibody-conjugates/phospho-stat5-tyr694-d47e7-xp-rabbit-mab-pe-conjugate/14603?srsltid=AfmBOooxZstdWJla_P2-MmiLASVyQKHvzzLT2QGOnTPc00nAYD6ZpCko

PE anti-p38 MAPK Phospho (Thr180/Tyr182) Recombinant Antibody Biolegend, Cat# 690254, Clone: A16016A.Rec; RRID:AB_3097635
https://www.biolegend.com/en-ie/products/pe-anti-p38-mapk-phospho-thr180-tyr182-recombinant-antibody-24934

Anti-Human CD3-FITC BD Biosciences Cat# 561802, Clone: HIT3a; RRID:AB_395745
https://www.bdbiosciences.com/en-us/products/reagents/flow-cytometry-reagents/research-reagents/single-color-antibodies-ruo/fitc-mouse-anti-human-cd3.561802

Anti-Human CD4-FITC BD Biosciences Cat# 555346, Clone: RPA-T4; RRID:AB_395751
https://www.bdbiosciences.com/en-us/products/reagents/flow-cytometry-reagents/research-reagents/single-color-antibodies-ruo/fitc-mouse-anti-human-cd4.555346

Anti-Human CD8-FITC BD Biosciences Cat# 555634, Clone: HIT8a; RRID:AB_395996
https://www.bdbiosciences.com/en-us/products/reagents/flow-cytometry-reagents/research-reagents/single-color-antibodies-ruo/fitc-mouse-anti-human-cd8.555634

Anti-Human CD14-FITC BD Biosciences Cat# 555397, Clone: M5E2; RRID:AB_395798
https://www.bdbiosciences.com/en-us/products/reagents/flow-cytometry-reagents/research-reagents/single-color-antibodies-ruo/fitc-mouse-anti-human-cd14.555397

Anti-Human CD15-FITC BD Biosciences Cat# 555401, Clone: HI98; RRID:AB_395801
https://www.bdbiosciences.com/en-us/products/reagents/flow-cytometry-reagents/research-reagents/single-color-antibodies-ruo/fitc-mouse-anti-human-cd15.555401

Anti-Human CD16-FITC BD Biosciences Cat# 555406, Clone: 3G8; RRID:AB_395806

https://www.bdbiosciences.com/en-us/products/reagents/flow-cytometry-reagents/research-reagents/single-color-antibodies-ruo/fitc-mouse-anti-human-cd16.555406

Anti-Human CD19-FITC BD Biosciences Cat# 555412, Clone: HIB19; RRID:AB_395812
https://www.bdbiosciences.com/en-us/products/reagents/flow-cytometry-reagents/research-reagents/single-color-antibodies-ruo/fitc-mouse-anti-human-cd19.555412

Anti-Human CD20-FITC BD Biosciences Cat# 555622, Clone: 2H7; RRID:AB_395988
https://www.bdbiosciences.com/en-us/products/reagents/flow-cytometry-reagents/research-reagents/single-color-antibodies-ruo/fitc-mouse-anti-human-cd20.555622

Anti-Human CD33-FITC BD Biosciences Cat# 555626, Clone: HIM3-4; RRID:AB_395992
https://www.bdbiosciences.com/en-us/products/reagents/flow-cytometry-reagents/research-reagents/single-color-antibodies-ruo/fitc-mouse-anti-human-cd33.555626

Anti-Human CD34-FITC BD Biosciences Cat# 555821, Clone: 581; RRID:AB_396150
https://www.bdbiosciences.com/en-us/products/reagents/flow-cytometry-reagents/research-reagents/single-color-antibodies-ruo/fitc-mouse-anti-human-cd34.555821

Anti-Human CD203C (NP4D6)-FITC Thermo Fisher Scientific Cat# MA5-28586, Clone: NP4D6; RRID:AB_2745545
https://www.thermofisher.com/antibody/product/CD203c-Antibody-clone-NP4D6-Monoclonal/MA5-28586

Anti-Human FcεRIα-FITC Biolegend Cat# 334608, Clone: AER-37 (CRA-1); RRID:AB_1227654
https://www.biolegend.com/en-ie/products/fitc-anti-human-fcepsilonrialpha-antibody-5059

Anti-Human CD56 (NCAM-1)-AF700 BD Biosciences Cat# 557919, Clone: B159; RRID:AB_396940
https://www.bdbiosciences.com/en-us/products/reagents/flow-cytometry-reagents/research-reagents/single-color-antibodies-ruo/alexa-fluor-700-mouse-anti-human-cd56-ncam-1.557919

Anti-Human CD56 (NCAM-1)-FITC BD Biosciences Cat# 562794, Clone: B159; RRID:AB_2737799
https://www.bdbiosciences.com/en-us/products/reagents/flow-cytometry-reagents/research-reagents/single-color-antibodies-ruo/fitc-mouse-anti-human-cd56-ncam-1.562794

Anti-Human CD56-BV421 BD Biosciences Cat# 562751, Clone: NCAM16.2; RRID:AB_2732054
https://www.bdbiosciences.com/en-us/products/reagents/flow-cytometry-reagents/research-reagents/single-color-antibodies-ruo/bv421-mouse-anti-human-cd56.562751

Anti-Human CD127-APC BD Biosciences Cat# 558598, Clone: HIL-7R-M21; RRID:AB_647113
https://www.bdbiosciences.com/en-us/products/reagents/flow-cytometry-reagents/research-reagents/single-color-antibodies-ruo/alexa-fluor-647-mouse-anti-human-cd127.558598

Anti-Human CD117-PE BD Biosciences Cat# 555714, Clone: YB5.B8; RRID:AB_396058
https://www.bdbiosciences.com/en-us/products/reagents/flow-cytometry-reagents/research-reagents/single-color-antibodies-ruo/pe-mouse-anti-human-cd117.555714

Anti-Human CD294 (CRTH2)-PE-Cy7 Biolegend Cat# 350118, Clone: BM16; RRID:AB_2562470
https://www.biolegend.com/en-ie/products/pe-cyanine7-anti-human-cd294-crth2-antibody-8815

Anti-Human NKp46-BV605 BD Bioscience Cat# 743710, clone: 9E2/NKp46; RRID:AB_2741689
https://www.bdbiosciences.com/en-us/products/reagents/flow-cytometry-reagents/research-reagents/single-color-antibodies-ruo/bv605-mouse-anti-human-cd335-nkp46.743710

7-AAD Staining Solution 2mL antibody BD Biosciences Cat# 559925, RRID:AB_2869266
https://www.bdbiosciences.com/en-us/products/reagents/flow-cytometry-reagents/research-reagents/single-color-antibodies-ruo/7-aad.559925

DAPI Solution BD Biosciences Cat# 564907, RRID:AB_2869624
https://www.bdbiosciences.com/en-us/products/reagents/flow-cytometry-reagents/research-reagents/single-color-antibodies-ruo/dapi-solution.564907

Violet Live Cell Caspase Probe BD Biosciences Cat# 565521, RRID: AB_2869682
https://www.bdbiosciences.com/en-us/products/reagents/flow-cytometry-reagents/research-reagents/single-color-antibodies-ruo/violet-live-cell-caspase-probe.565521

Annexin V-PE Biolegend Cat# 640947, RRID: NO
https://www.biolegend.com/en-ie/products/pe-annexin-v-8145

Anti-Human TNF BV650 Biolegend Cat# 502938, Clone: MAb11; RRID: AB_2561355
https://www.biolegend.com/en-ie/products/brilliant-violet-650-anti-human-tnf-alpha-antibody-7680

Anti-Human IFN-γ -BV421 Biolegend Cat# 506538 , Clone: B27; RRID: AB_2801097
https://www.biolegend.com/en-ie/products/brilliant-violet-421-anti-human-ifn-gamma-antibody-17133

Phospho-NF-κB p65 (Ser536) (93H1) Rabbit mAb (PE Conjugate) Cell signaling Technology Cat# 5733, Clone: 93H1; RRID: AB_10706937
https://www.cellsignal.com/products/antibody-conjugates/phospho-nf-kb-p65-ser536-93h1-rabbit-mab-pe-conjugate/5733

PE anti-NF-κB p65 Antibody Biolegend Cat# 653004, Clone: 14G10A21; RRID: AB_2562768
https://www.biolegend.com/en-ie/products/pe-anti-nf-kappab-p65-antibody-9073

BD Horizon™ BUV395 Mouse Anti-Ki-67 BD Biosciences Cat# 564071, Clone: B56; RRID:AB_2738577
https://www.bdbiosciences.com/en-us/products/reagents/flow-cytometry-reagents/research-reagents/single-color-antibodies-ruo/buv395-mouse-anti-ki-67.564071

BD Horizon™ BV421 Rabbit Anti-Active Caspase-3 BD Biosciences Cat# 570786, Clone: C92-605.rMAb; RRID: NO
https://www.bdbiosciences.com/en-us/products/reagents/flow-cytometry-reagents/research-reagents/single-color-antibodies-ruo/bv421-rabbit-anti-active-caspase-3.570786

Rat IgG2a kappa Isotype Control (eBR2a) Thermofisher Cat# 16-4321-82, clone: eBR2a; RRID: AB_470156
https://www.thermofisher.com/antibody/product/Rat-IgG2a-kappa-clone-eBR2a-Monoclonal/16-4321-82

CD335 (NKp46) Monoclonal Antibody (29A1.4) Thermofisher Cat# 16-3351-81, Clone: 29A1.4; RRID: AB_1724164
https://www.thermofisher.com/antibody/product/CD335-NKp46-Antibody-clone-29A1-4-Monoclonal/16-3351-81

NKp46 Polyclonal Antibody Thermofisher Cat# PA5-46986, clone: Polyclonal; RRID: AB_2606904
https://www.thermofisher.com/antibody/product/NKp46-Antibody-Polyclonal/PA5-46986

Ultra-LEAF™ Purified anti-mouse CD314 (NKG2D) Antibody Biolegend Cat# 115713, Clone: C7; RRID: AB_2832393
https://www.biolegend.com/en-ie/products/ultra-leaf-purified-anti-mouse-cd314-nkg2d-antibody-19074

BD Horizon™ BV510 Mouse anti-BrdU BD Biosciences Cat# 563445, Clone: 3D4; RRID: AB_2738210
https://www.bdbiosciences.com/en-us/products/reagents/flow-cytometry-reagents/research-reagents/single-color-antibodies-ruo/bv510-mouse-anti-brdu.563445

Eukaryotic cell lines

Policy information about cell lines and Sex and Gender in Research

Cell line source(s)	The mouse AML cell line C1498 (Cat# TIB-49) and human AML cell line THP-1 (TIB-202) are purchased from the American Type Culture Collection (ATCC). The human AML cell line MOLM13 (ACC-554) was purchased from the Leibniz Institute DSMZ - German Collection of Microorganisms and Cell Cultures GmbH.
Authentication	Cell lines were authenticated according ATCC cell line authentication test recommendations.
Mycoplasma contamination	All cell lines were routinely tested for the absence of Mycoplasma using the MycoAlert Plus Mycoplasma Detection Kit from Lonza (Cat# M7006). Cell line is mycoplasma-free.
Commonly misidentified lines (See ICLAC register)	No commonly misidentified cell lines were used in this study.

Animals and other research organisms

Policy information about studies involving animals; ARRIVE guidelines recommended for reporting animal research, and Sex and Gender in Research

Laboratory animals	C57BL/6J and Ncr1gf/gfp (C57BL/6J background) mice were purchased from the Jackson laboratory. All mice were used at 6-12 weeks of age.
Wild animals	No wild animals were used in this study.
Reporting on sex	Both male and female mice were used in all experiments.
Field-collected samples	The study did not involve samples collected from the field.
Ethics oversight	Mouse care and experimental procedures were performed in accordance with federal guidelines and protocols approved by the Institutional Animal Care and Use Committee at City of Hope under protocol numbers 18108 and 20003.

Note that full information on the approval of the study protocol must also be provided in the manuscript.

Plants

Seed stocks	N/A
Novel plant genotypes	N/A
Authentication	N/A

Flow Cytometry

Plots

Confirm that:

- [x] The axis labels state the marker and fluorochrome used (e.g. CD4-FITC).
- [x] The axis scales are clearly visible. Include numbers along axes only for bottom left plot of group (a 'group' is an analysis of identical markers).
- [x] All plots are contour plots with outliers or pseudocolor plots.
- [x] A numerical value for number of cells or percentage (with statistics) is provided.

Methodology

Sample preparation	Cells were collected, washed with 1 % FBS + DPBS, stained with different fluorescence-conjugated antibodies at 4°C for 30 min, 1 % FBS + DPBS twice, and then analyzed using BD LSRFortessaTM X20 and BD FACSAriaTM Fusion.
Instrument	Cells were either analyzed on BD LSRFortessaTM X20 or sorted by BD FACSAriaTM Fusion.
Software	Flow Cytometry data were analyzed by FlowJo V10 (Treestar).
Cell population abundance	The purity of sorted cells was detected via D FACSAriaTM Fusion and samples with purity >90% were used.
Gating strategy	ILC1s from human peripheral blood were identified using surface staining with a live/dead cell viability cell staining kit and the following monoclonal antibodies: lineage (anti-CD3, anti-CD4, anti-CD8, anti-CD14, anti-CD15, anti-CD16, anti-CD19, anti-CD20, anti-CD33, anti-CD34, anti-CD203c, anti-FceRI), anti-CD56, anti-CD127, anti-CRTH2, and anti-c-Kit. ILC1s or NK cells from mice were identified using live/dead cell viability dyes and the following monoclonal antibodies: lineage (anti-CD3 and anti-CD19), anti-NK1.1, anti-NKp46, anti-CD49b, and anti-CD49a. Human ILC1s were gated by Lin-CD56-CD127+CRTH2-c-Kit-. Mouse ILC1s were gated by Lin-NK1.1+ NKp46+ CD49b-CD49a+. Mouse NK cells were gated by Lin-NK1.1+ NKp46+ CD49b +CD49a-.

- [x] Tick this box to confirm that a figure exemplifying the gating strategy is provided in the Supplementary Information.
